# Spermidine Suppresses Age-Associated Memory Impairment by Preventing Adverse Increase of Presynaptic Active Zone Size and Release

**DOI:** 10.1371/journal.pbio.1002563

**Published:** 2016-09-29

**Authors:** Varun K. Gupta, Ulrike Pech, Anuradha Bhukel, Andreas Fulterer, Anatoli Ender, Stephan F. Mauermann, Till F. M. Andlauer, Emmanuel Antwi-Adjei, Christine Beuschel, Kerstin Thriene, Marta Maglione, Christine Quentin, René Bushow, Martin Schwärzel, Thorsten Mielke, Frank Madeo, Joern Dengjel, André Fiala, Stephan J. Sigrist

**Affiliations:** 1 Institute for Biology/Genetics, Freie Universität Berlin, Berlin, Germany; 2 NeuroCure, Charité, Berlin, Germany; 3 Georg-August-Universität Göttingen, Molecular Neurobiology of Behavior, Göttingen, Germany; 4 Max Planck Institute of Psychiatry, Munich, Germany; 5 Centre for Systems Biological Analysis, University of Freiburg, Freiburg, Germany; 6 Max Planck Institute for Molecular Genetics, Berlin, Germany; 7 Institute for Molecular Biosciences, NAWI Graz, University of Graz, Graz, Austria; 8 BioTechMed Graz, Graz, Austria; University of Massachusetts Medical School, UNITED STATES

## Abstract

Memories are assumed to be formed by sets of synapses changing their structural or functional performance. The efficacy of forming new memories declines with advancing age, but the synaptic changes underlying age-induced memory impairment remain poorly understood. Recently, we found spermidine feeding to specifically suppress age-dependent impairments in forming olfactory memories, providing a mean to search for synaptic changes involved in age-dependent memory impairment. Here, we show that a specific synaptic compartment, the presynaptic active zone (AZ), increases the size of its ultrastructural elaboration and releases significantly more synaptic vesicles with advancing age. These age-induced AZ changes, however, were fully suppressed by spermidine feeding. A genetically enforced enlargement of AZ scaffolds (four gene-copies of BRP) impaired memory formation in young animals. Thus, in the *Drosophila* nervous system, aging AZs seem to steer towards the upper limit of their operational range, limiting synaptic plasticity and contributing to impairment of memory formation. Spermidine feeding suppresses age-dependent memory impairment by counteracting these age-dependent changes directly at the synapse.

## Abstract 2

Feeding spermidine to *Drosophila* protects them from age-dependent memory impairment by suppressing structural and functional alterations in presynaptic performance.

## Author Summary

Neurons communicate by sending impulses, in the form of secretion of neurotransmitters, across small spaces called synapses. It is these synapses that undergo structural and functional changes during formation and retrieval of memories. Though alterations in synaptic performance are believed to accompany aging, the causal relationship between age-dependent memory impairment and synaptic changes remains largely unknown. Using the fly *Drosophila melanogaster* as a model, we found that feeding them spermidine—a polyamine compound—suppresses age-induced decline in olfactory memory, providing us with a tool to further decipher mechanisms associated with age-dependent memory impairment. In this study, we investigated the relationship between synaptic changes and age-dependent memory impairment by studying the olfactory circuitry. We observed an age-related increase in the levels of the synaptic proteins Bruchpilot and Rim-binding protein, which caused an enlargement of the presynaptic active zone—the complex of proteins that mediate neurotransmitter release—and enhanced synaptic transmission. Interestingly, feeding of spermidine was sufficient to abolish these age-associated presynaptic changes, further emphasizing the relationship between presynaptic performance and age-dependent memory impairment. Furthermore, flies engineered to express an excess of the core active zone protein Bruchpilot showed a premature impairment in memory formation in young flies. Based on our data, aging plausibly steers the synapses towards the upper limit of their operational range, limiting synaptic plasticity and contributing to impairment of memory formation.

## Introduction

Age-dependent memory impairment (AMI), which is associated with both psychiatric and neurodegenerative disorders, starts in midlife and worsens with advancing age, suggesting that the greatest driving factor is age itself. The lack of effective treatments that prevent, halt, or reverse the condition is contributing to a diminishing quality of life for many senior citizens. Therefore, animal models that allow one to monitor physiological changes across their lifespan and to test for a causal character of age-induced changes might be helpful in exploring the mechanistic basis of AMI. *D*. *melanogaster*, with its short lifespan of around 60 d and advanced molecular genetic tools, provides an efficient experimental model to unravel mechanisms underlying AMI. Additionally, the olfactory nervous systems of insects and mammals exhibit many similarities, suggesting that the mechanisms for olfactory learning may be shared [1]. Moreover, aversive short-, intermediate-, and long-term olfactory memories have been found to be subject to age-induced decline in *Drosophila*, with an onset at about 10 d of age and plateaus at about 30 d of age [2–6]. Notably, we recently found a simple dietary supplementation of spermidine, a polyamine that specifically protects from AMI in *Drosophila*.

External stimuli are believed to be represented in the brain as spatiotemporal patterns of neural activity within a set of neuronal connections. Changes in synaptic communication (“plasticity”) within certain neuron populations are meant to ultimately encode behavioral adaptations such as learning and memory. Thus, dysfunctioning of synaptic plasticity might well be relevant to age-dependent deterioration of learning and memory [7,8]. One of the fundamental problems of studying AMI, however, is the inability to differentiate causative changes from adaptive or protective changes. Moreover, the brain undergoes changes at multiple levels with advancing age, including alterations in circuits, individual neurons, and single synapses, further complicating the situation [8]. Nonetheless, recent work has linked AMI to subtle synaptic alterations in the hippocampus and other cortical brain areas, rather than to the loss of neurons [7,9]. At the same time, the age-associated modulation of molecular entities underlying learning and memory that define and change synapse function remain poorly understood. Therefore, we set out to determine the role of age-induced changes in the organization and function of synapses in AMI, using dietary supplementation with spermidine as a tool to identify synaptic changes that can potentially contribute to AMI.

To accomplish this, we analyzed age-induced changes in the ultrastructural, molecular, and functional organization of synapses within the olfactory system of flies by comparing aged flies fed with normal food to aged flies fed with spermidine-supplemented food. We found that aging is associated with an increase in the average size of active zone (AZ) scaffolds, structures recently shown to scale with synaptic vesicle (SV) release. Consistent with this, optophysiological analysis showed that more SVs are released in response to natural odor stimuli in aged flies. Interestingly, these age-associated changes were suppressed by spermidine feeding, indicating that these changes might be causally relevant to AMI. In fact, genetic manipulation provoking an increase of T-bar size in young animals was sufficient to induce a premature decline in memory performance. We suggest that a cumulative increase in the size and function of presynaptic AZ scaffolds might reduce the operational range of synaptic plasticity processes, and thus, hamper the formation of new memories with age. Additionally, levels of postsynaptic neurotransmitter receptors and postsynaptic Ca^2+^ signals remained largely unaffected with age, suggesting that homeostatic adaptations might be involved in increasing the threshold for memory formation with advancing age.

## Results

It is known that the ability to acquire new memories declines with advancing age. Based on previous study [10], one plausible explanation for this observation might be the increase in the threshold required for memory formation with age. In fact, when we analyzed olfactory conditioning in aged flies (30-d-old flies or 30d) we found that greater number of exposures to the unconditioned stimulus in order to attain saturated levels of memory scores, which, however, never reached the same maximal learning scores found in young flies (3-d-old flies or 3d), indicating that the dynamic range of memory formation is altered with advancing age (S1 Fig). Multiple lines of evidence suggest that presynaptic plasticity processes are responsible for forming olfactory associative memory in *Drosophila* [11–13]. Therefore, we set out to determine the role of age-induced changes in the organization and function of synapses in AMI, using dietary supplementation with spermidine as a tool to identify synaptic changes that can potentially contribute to AMI.

### Age-Induced Increase of Odor-Driven Vesicle Release Is Suppressed by Spermidine Feeding

In order to identify synaptic mechanisms plausibly contributing to AMI, we used opto-physiological assays to characterize overall neuronal responses in synaptic terminals of live intact flies. For these experiments, we focused on projection neuron (PN) to kenyon cell (KC) synapses within the mushroom body calyx of the olfactory system for two reasons: first, aversive olfactory learning involves coincidence detection of a conditioned stimulus (odor) with an unconditioned stimulus (electric shock), causing changes in the odor-specific synaptic activity of second order PNs and third order mushroom body KCs [1,14]; second, the superficial position of the calyx within the fly brain enabled us to perform efficient optical analysis [15], since sensor signals could be retrieved from discrete synaptic bouton areas.

We started by expressing cytosolic GCamp3.0 in the PNs (using GH146-Gal4) and found the basal expression of GCamp3.0 to remain largely unchanged with age (S2A Fig). Next, we monitored the PN boutons for intracellular Ca^2+^ responses to two odors typically used for olfactory conditioning, 3-Octanol (3-Oct) and 4-methylcyclehexanol (MCH), through two-photon microscopy. Similar to our previous observations [3], we found no significant difference in the amplitude or time course of cytosolic GCamp3.0 signals of young (3d) and aged (30d) animals (S3 Fig). Thus, in the context of odor information processing, odor-evoked action potential frequency or presynaptic Ca^2+^ influx remained rather unaffected by the age of the animal.

**Fig 1:**
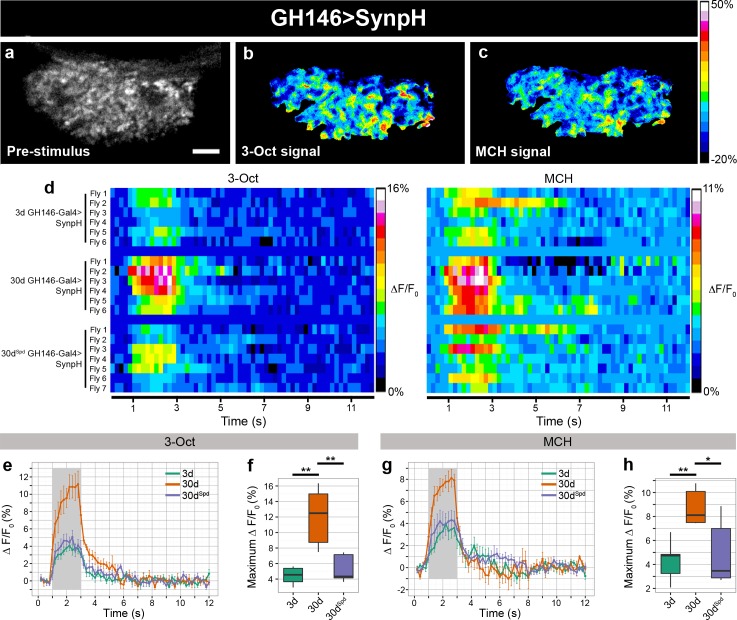
Imaging of SynpH at PN-to-KC synapses to measure odor-evoked SV release. (a) SynpH expressed in PN boutons and imaged within the calyx neuropil (GH146 > SynpH). Scale bar: 10 μm. (b–c) False color-coded image of the SynpH activity within the presynaptic terminals of PNs in response to 3-Oct and MCH shown in (a). Warm colors indicate high levels, and cold colors indicate low levels or no SynpH activity. The color scale on the right indicates changes in fluorescence (*ΔF/*F in %). (d) Odor-evoked release of SVs, measured by changes in fluorescence of SynpH of individual flies over time shown as false colors in presynaptic terminals of PN in the calyx region. The left panel is in response to the odorant 3-Oct and the right panel is in response to MCH (*n* = 6–7 flies). (e) Time course of SynpH activity induced by 3-Oct in the presynaptic terminals of PNs within the calyx neuropil of 3d, 30d, and 30d^Spd^ animals (SynpH response averaged across three odor exposures from 6–7 flies). (f) Maximum change in SynpH fluorescence (ΔF*/F* in %) in response to 3-Oct within the presynaptic terminals of PN boutons of 3d, 30d, and 30d^Spd^ flies (SynpH response averaged across three odor exposures from 6–7 flies; Kruskal-Wallis test with Dunn’s multiple comparison test, *p*-values were subject to Bonferroni correction). (g) Time course of SynpH activity induced by MCH in the presynaptic terminals of PNs within the calyx region of 3d, 30d, and 30d^Spd^ animals (SynpH response averaged across three odor exposures from 6–7 flies) (h) Maximum change in SynpH fluorescence (ΔF*/F* in %) in response to MCH within the presynaptic terminals of PN boutons of 3d, 30d, and 30d^Spd^ flies (SynpH response averaged across three odor exposures from 6–7 flies; Kruskal-Wallis test with Dunn’s multiple comparison test, *p*-values were subject to Bonferroni correction). * *p* < 0.05, ** *p* < 0.01, ns = not significant, *p* ≥ 0.05. Underlying data is shown in S1 Data.

Next, we asked whether the release of SVs was altered with advancing age and analyzed the odor-driven SVs release. To this end, we used SynaptopHluorin (SynpH), a pH-sensitive green fluorescent protein (GFP) fused to the luminal side of the SV membrane protein Synaptobrevin (Syb) [16]. SynpH is nonfluorescent at the acidic pH inside SVs; however, when SVs are released, SynpH is exposed to the neutral extracellular space, and the presynaptic terminal becomes brightly fluorescent. Following endocytosis, SVs become reacidified, and the cycle can start again [17]. SynpH was expressed within PNs, and the release of SVs in response to two odors was monitored, again, at PN-to-KC synapses (Fig 1A–1H). We found a profound increase in the amplitude of SynpH signals in aged (30d) animals when compared to young (3d) flies (Fig 1A–1H). In contrast, spermidine administration to 30d flies prevented this age-dependent increase of odor-induced SynpH signals (30d^Spd^; Fig 1A–1H). Alterations in the endocytotic clearance of newly released SVs might, per se, explain the increase in SynpH signals observed; however, the decay constants of the poststimulus SynpH signal remained essentially unchanged with aging (S4 Fig), indicating that the endocytic clearance cannot be responsible for the difference in odor-driven SynpH signals observed in aged animals. In addition, neither the basal expression of SynpH before odor stimulation nor the maximal SynpH signal determined by high-molar KCl treatment showed systematic differences between young and aged cohorts (S2B and S5 Figs). These experiments, thus, indicate that the exocytosis of SVs underlies the increase in SynpH response with advancing age.

In addition to measuring the SV release at the PN presynaptic terminals within calyx, we also measured odor-evoked changes within the axonal projections of KCs within the mushroom body horizontal lobes by expressing SynpH using mb247-Gal4. Though relative signals were smaller (when signal was normalized to the whole mushroom body horizontal lobe), likely reflecting the well-documented sparse odor coding of KCs [18], we still observed a substantially higher amplitude of SynpH signals in aged (30d) than in young (3d) flies, and, again, spermidine administration (30d^Spd^) protected from this age-dependent increase (S6 Fig). Thus, two major neuron populations of the olfactory system—PNs and KCs, showed an increase in odor-evoked fluorescence changes in response to odor stimuli, indicating higher release of SVs in aged animals.

### Spermidine Feeding Specifically Blocks Age-Induced Increases of Core AZ Components

**Fig 2:**
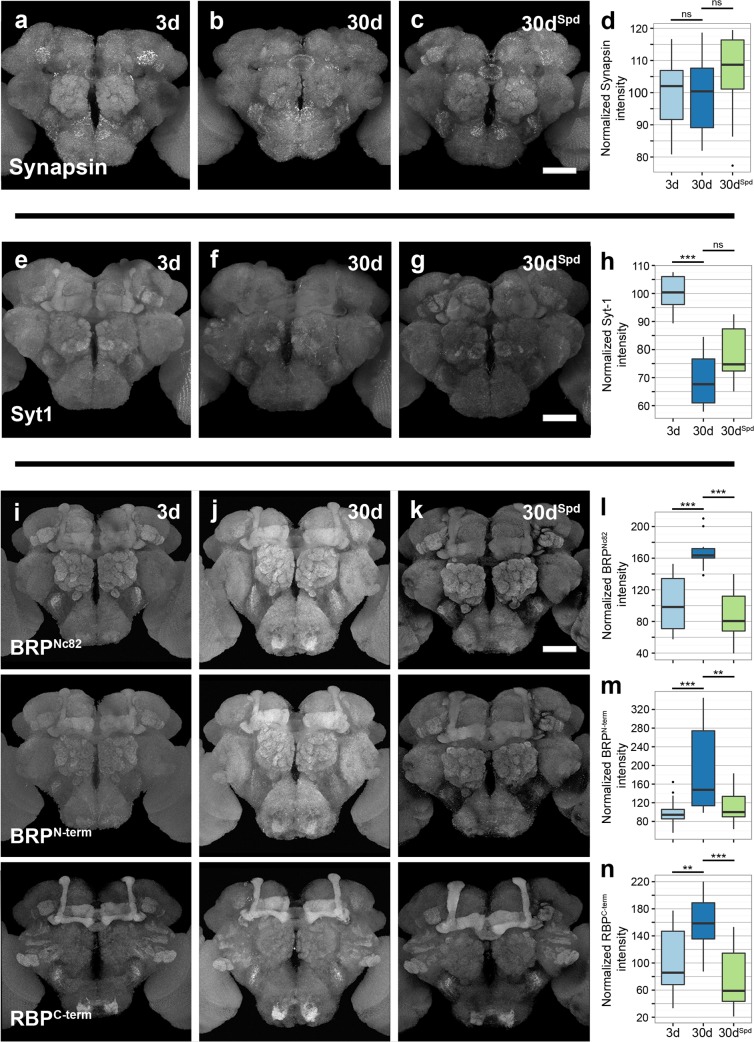
Spermidine feeding suppresses age-associated increase in BRP and rim-binding protein (RBP) levels. (a–c) Adult brains 3d and 30d *w*^*1118*^ flies, together with 30d^Spd^ *w*^*1118*^ flies immunostained for Synapsin. Scale bar: 50 μm. (d) Quantification of Synapsin intensity within the central brain region normalized to 3d flies (*n* = 9–10 independent brains; Kruskal-Wallis test). (e–g) Adult brains of 3d and 30d *w*^*1118*^ flies, together with 30d^Spd^ *w*^*1118*^ flies immunostained for Synaptotagmin-1 (Syt-1). Scale bar: 50 μm. (h) Quantification of signal intensity of Syt-1 in the central brain region normalized to 3d flies (*n* = 8–9 independent brains; Kruskal-Wallis test with Dunn’s multiple comparison test, *p*-values were subject to Bonferroni correction). (i–k) Adult brains of 3d, 30d *w*^*1118*^, and 30d^Spd^ *w*^*1118*^ flies immunostained for BRP (using Nc82 and N-terminal antibodies) and RBP. Scale bar: 50 μm (l–n) Quantification of BRP (using Nc82 and N-terminal antibodies) and RBP intensities within the central brain region normalized to 3d flies (*n* = 14–18 independent brains; Kruskal-Wallis test with Dunn’s multiple comparison test, *p*-values were subject to Bonferroni correction). ** *p* < 0.01, *** *p* < 0.001, ns = not significant, *p* ≥ 0.05. Underlying data is shown in S1 Data.

Since Ca^2+^ influx into presynaptic terminals was apparently not responsible for the profound age-induced increase in SV release, presynaptic mechanisms downstream of Ca^2+^ signaling might be involved. In order to address the molecular and cellular basis of this age-associated increase in SV release, we started by analyzing proteins directly associated with SVs: Synapsin, Syb, and Synaptotagmin-1. Synapsin is a SV-associated phosphoprotein important for controlling the number of SVs available for release [19], and Syb is a core component of SNARE complex that drives the exocytosis of SVs [20,21]. We found the levels of Synapsin as well as Syb to remain unchanged with advancing age (comparing aged flies: 30-days old or 30d with young flies: 3d), regardless of spermidine feeding (30d^Spd^; Fig 2A–2D and S7 Fig). Synaptotagmin-1 is a vesicular protein with a central role as a Ca^2+^ sensor for SNARE-dependent SV fusion [22]. Synaptotagmin-1 decreased slightly with age, and feeding with spermidine had no discernable influence on this age-dependent change (Fig 2E–2H), indicating that these moderate changes are seemingly not associated with AMI.

The release of neurotransmitters is a sophisticated process that requires SVs to be in close vicinity to voltage-gated Ca^2+^ channels, and this precise spacing is orchestrated by interplay among several proteins that form the AZ scaffold [23,24]. In flies, the ELKS-family protein Bruchpilot (BRP) is an essential building block of the AZ scaffold and is needed to effectively cluster Ca^2+^ channels as well as regulate the release of SVs [25–28]. When whole-mount brains were stained for BRP using two different antibodies (BRP^Nc82^ and BRP^N-term^), we observed a substantial increase in the levels of BRP with advancing age (Fig 2I, 2J, 2L and 2M). Similarly, Rim-binding protein (RBP) [29], another structurally and functionally important component of the AZ scaffold, was found to be significantly increased in brains of 30d flies compared to 3d animals (Fig 2I, 2J and 2N). Furthermore, flies analyzed at shorter intervals throughout their lifetime exhibited a progressive increase in the levels of both BRP and RBP (S8 Fig). Notably, the age-dependent increase in BRP and RBP signals was suppressed in aged flies fed with spermidine (30d^Spd^; Fig 2I–2N).

The staining efficacy could potentially be influenced by the sheer age of the tissue, e.g., due to differences in antibody penetration. To rule this out, flies expressing a GFP-tagged genomic BRP construct (rescuing the lethal *brp* null mutant [28]) were aged on normal food or food supplemented with spermidine. We found the endogenous GFP signals to be significantly increased in 30d flies in comparison to 3d flies, while feeding with spermidine again prevented this age-related increase (S9 Fig).

Since the AZ scaffold has previously been reported to effectively cluster Ca^2+^ channels [26–28], we asked whether the age-associated increase in levels of core AZ-proteins might influence synaptic levels of Ca^2+^ channels. To address this, we expressed a GFP-labeled genomic construct of α1 subunit Cacophony (Cac), which is the only representative of the mammalian Ca_v_2.1/2.2 family present in *Drosophila* [28], and stained the flies for GFP and BRP. We found the levels of Cac (quantified using an antibody against GFP) to remain unchanged with aging (S10 Fig). Besides its role in Ca^2+^ channel clustering, the AZ scaffold has been suggested to create a stereotypic arrangement that defines SV release slots by clustering SV release machinery [28]. In fact, the levels of Unc13, a protein essential for priming SVs by rendering them fusion-competent [24], were also increased in brains of 30d flies compared to 3d flies (S11 Fig). Again, spermidine administration suppressed this age-dependent increase (30d^Spd^; S11 Fig). Taken together, our data suggest that synaptic levels of core AZ scaffold proteins, BRP and RBP, as well as the levels of critical release factor Unc-13 increased with advancing age.

### Age-Induced Enlargement of AZ Scaffolds Is Suppressed by Spermidine Feeding

Next, we asked whether the increase of both BRP and RBP labeling in aged brains reflects an increase in the number of AZs or just the increase in local amounts of these proteins at individual AZs. To resolve this, we performed ultrastructural analysis on PN-to-KC synapses within the mushroom body calyx. In contrast to presynaptic terminals of KCs, presynaptic PN terminals within the calyx exhibit a well-defined morphology [30,31], by which synapse types can be reliably identified in EM micrographs. Moreover, the superficiality of the calyx enabled us to perform stimulation emission depletion microscopy (STED) analysis (see below).

**Fig 3:**
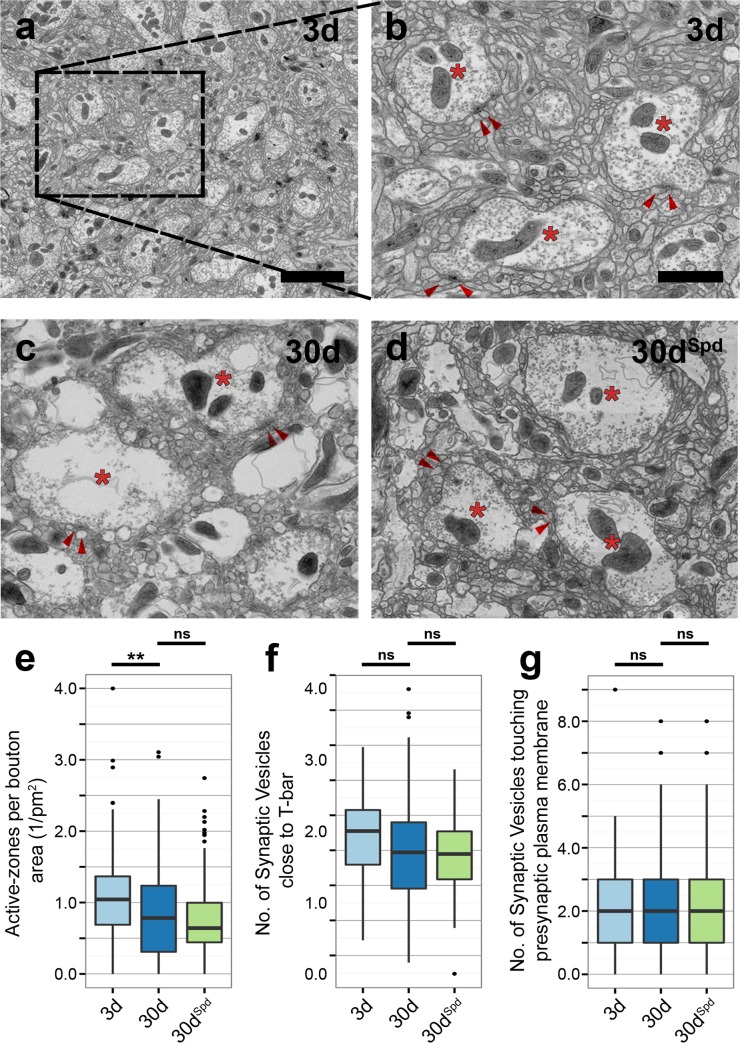
Ultrastructural analysis of PN-to-KC synapses within the mushroom body calyx. (a) Overview of the calyx neuropil, obtained by amalgamation of several images over a whole calyx cross-section of a 3d *w*^*1118*^ fly. Scale bar: 10 μm. (b–d) Higher magnification of PN boutons and dendritic claws of KCs within the calyx of 3d, 30d, and 30d^Spd^ *w*^*1118*^ flies. Scale bar: 2 μm. The asterisk indicates the PN bouton, and the arrowhead indicates the dendritic claws of KCs. (e) Quantification of AZs normalized to bouton area (1/pm^2^) (total of 95–103 boutons across three independent animals, with at least 25 boutons per animal; Kruskal-Wallis test with Dunn’s multiple comparison test, *p*-values were subject to Bonferroni correction). (f) Quantification of total SVs within a shell of 150 nm surrounding the AZ scaffold (total of 92–100 electron-micrographs across four independent animals, with at least 20 electron-micrographs per animal; Kruskal-Wallis test with Dunn’s multiple comparison test, *p*-values were subject to Bonferroni correction). (g) Quantification of SVs touching the presynaptic plasma membrane (total of 92–100 electron-micrographs across four independent animals, with at least 20 electron micrographs per animal; Kruskal-Wallis test with Dunn’s multiple comparison test, p-values were subject to Bonferroni correction). * *p* < 0.05, ** *p* < 0.01, *** *p* < 0.001, ns = not significant, *p* ≥ 0.05. Underlying data is shown in S1 Data.

In order to allow for an unbiased quantification, we applied automated data collection to acquire more than a thousand transmission electron microscopic images covering nearly a whole calyx cross-section, which were then “stitched” together into a single high-magnification image (see Materials and Methods). As described previously [30], PN boutons could be easily identified, and light-colored boutons containing clear-core SVs were used for analysis. We recognized that plasma membranes between cellular elements were less aligned, with an increase in extracellular spacing between cellular elements, in aged (30d) flies when compared to young (3d) flies (S12 Fig). Spermidine feeding appeared to substantially alleviate this age-related change (S12 Fig). Driven by the finding that SV release is increased with age, we decided to analyze the AZs within PN boutons. We found aged animals (30d) to display reduced numbers of AZs per unit bouton-area in comparison to 3d flies, with no apparent influence of spermidine feeding on this age-dependent decline (Fig 3A–3E). The density of SVs in proximity to the AZ scaffold appeared unchanged in aged flies (30d as well as 30d^Spd^), when compared to young flies (3d; Fig 3F). Additionally, the number of SVs docked at the AZ plasma membrane appeared essentially unaltered with advancing age (Fig 3G).

**Fig 4:**
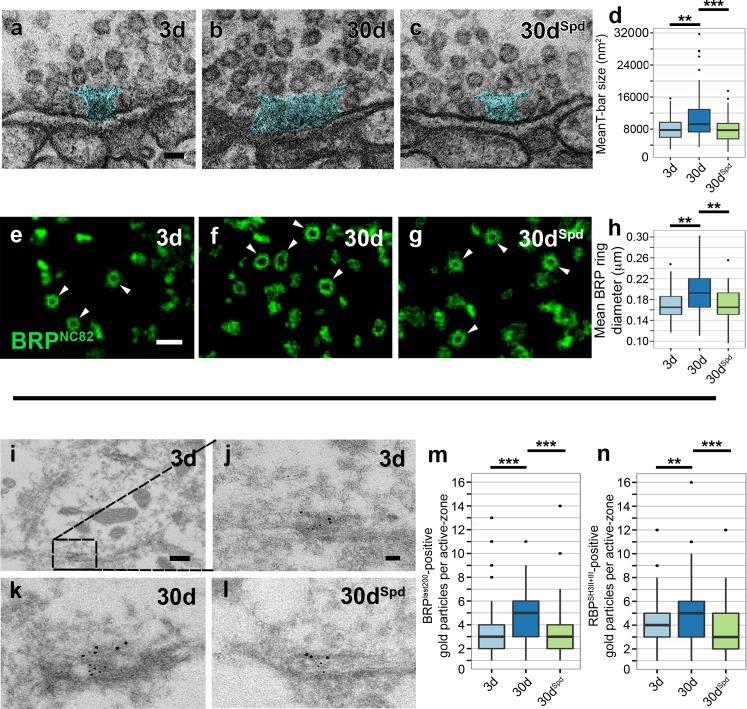
High-resolution analysis of PN-to-KC synapses within the mushroom body calyx shows increase in T-bar size. (a–c) Electron micrographs of calyx region of 3d, 30d, and 30d^Spd^ *w*^*1118*^ animals showing presynaptic specializations in blue (T-bars) at the PN-to-KC synapses. Scale bar: 50 nm. (d) Quantification representing the average T-bar size in 3d, 30d, and 30d^Spd^ animals (*n* = 92–100 electromicrographs across four independent animals, with at least 20 T-bars per animal; Kruskal-Wallis test with Dunn’s multiple comparison test, *p*-values were subject to Bonferroni correction). (e–g) STED images of BRP spots reveal ring-shaped structures (arrowheads) within the calyx of 3d, 30d, and 30d^Spd^ *w*^*1118*^ flies. Scale bar: 500 nm. (h) Comparison of BRP-spot diameter between 3d, 30d, and 30d^Spd^ flies (total of 94–112 BRP rings across 15 independent animals, with at least 5 BRP rings per animal; Kruskal-Wallis test with Dunn’s multiple comparison test, *p*-values were subject to Bonferroni correction). (i) Electron micrographs of PN bouton within the calyx region of 3d *w*^*1118*^ flies. Scale bar: 200 nm. (j–l) Higher magnification of AZ within PN bouton immunostained for BRP (large gold particles) and RBP (small gold particles) of 3d, 30d, and 30d^Spd^ *w*^*1118*^ flies. Scale bar: 50 nm. (m) Quantification of BRP-positive gold particles per T-bar (total of 94–108 individual T-bars across three independent animals, with at least 25 T-bars per animal; Kruskal-Wallis test with Dunn’s multiple comparison test, *p*-values were subject to Bonferroni correction). (n) Quantification of RBP-positive gold particles per T-bar (total of 94–108 individual T-bars across three independent animals, with at least 25 T-bars per animal; Kruskal-Wallis test with Dunn’s multiple comparison test, *p*-values were subject to Bonferroni correction). * *p* < 0.05, ** *p* < 0.01, *** *p* < 0.001, ns = not significant, *p* ≥ 0.05. Underlying data is shown in S1 Data.

The AZ scaffold exhibits an electron-dense structure in electron microscopy (EM), and due to its T-shaped structure in *Drosophila*, this scaffold is often referred to as a T-bar [24,26,27]. We found the average size of the T-bars to be significantly increased in 30d animals in comparison to 3d flies (Fig 4A–4D). Feeding flies with spermidine suppressed this age-induced increase in T-bar size (30d^Spd^; Fig 4A–4D). We have previously introduced STED in the analysis of AZ suborganization [26–28]. At peripheral neuromuscular synapses of *Drosophila* larvae, STED allowed us to unmask the “nano-architecture” of AZs where BRP and RBP organize a scaffold that provides slots for SV release and concentrates Ca^2+^ channels in the AZ center [28,29]. When planar AZs were imaged using the BRP C-terminal epitopes at neuromuscular synapses, they display a ring-shaped structure whose diameter correlated with the EM-derived physical size of individual T-bar/AZ scaffold [32]. We applied STED to PN-to-KC synapses of the calyx and found ring-like BRP structures at planar-oriented AZs (S13 Fig). Subsequently, the analysis of these STED images revealed an increase in the ring diameter of BRP spots with advancing age, while spermidine treatment was able to suppress this age-associated increase (Fig 4E–4H). Finally, we performed coimmuno-EM labeling against BRP and RBP on calycal slices. The number of gold particles positive for BRP as well as RBP was found to increase in aged flies (30d) in comparison to both young (3d) flies and aged flies fed with spermidine (30d^Spd^; Fig 4I–4N). Taken together, the morphological EM, immuno-EM, and STED analysis consistently show that aged animals display larger AZ scaffolds, plausibly due to an increase in local amounts of the critical scaffold components: BRP and RBP.

Recent in vivo analysis of larval *Drosophila* neuromuscular junctions has shown that the local amounts of BRP at a given AZ scale directly with the probability of evoked SV release [33–37]. Consistent with these studies, we found SV release to increase and AZ scaffolds to enlarge with age, while importantly both these age-related changes were suppressed by dietary supplementation with spermidine. Therefore, we next wanted to determine the influence of these synaptic changes on olfactory memory formation.

### “Early” Memory Impairment after Genetically Enforced Enlargement of AZ Scaffolds

**Fig 5:**
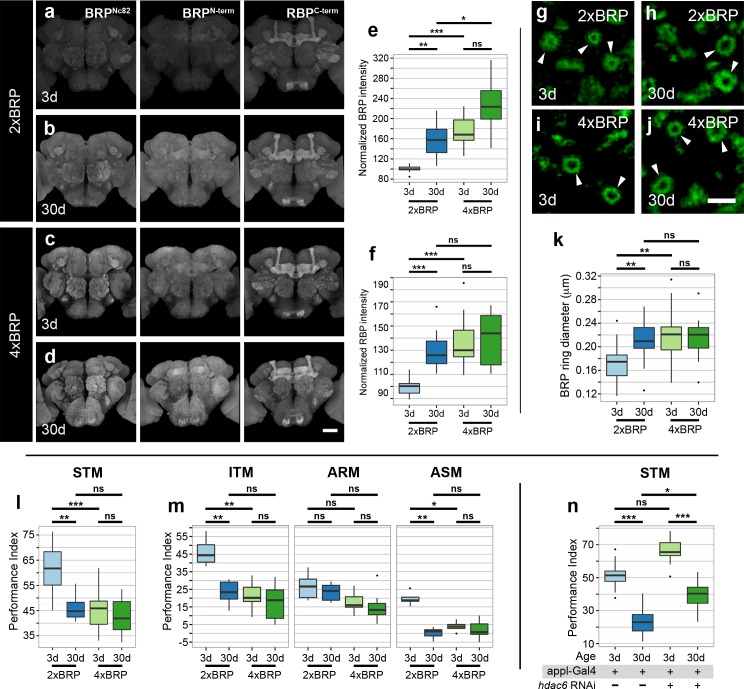
Increase in levels of core AZ proteins BRP and RBP leads to early memory impairment. (a–d) Adult brains of 3d and 30d flies expressing 4xBRP together with age-matched controls *brp* (2xBRP), immunostained for BRP (using Nc82 and BRP N-terminal antibody) and RBP. Scale bar: 50 μm. (e, f) Quantification of BRP (using N-terminal antibody) as well as RBP intensity within the central brain region normalized to 3d flies (*n* = 12–13 independent brains; Kruskal-Wallis test with Dunn’s multiple comparison test, *p*-values were subject to Bonferroni correction). (g–j) STED images of BRP label within the calyx region of 3d and 30d flies expressing 4xBRP as well as 2xBRP. Ring-shaped structures are indicated (arrowheads). Scale bar: 500 nm. (k) Quantification of BRP ring diameter in 3d and 30d 4xBRP flies along with age-matched 2xBRP flies (total of 47–68 BRP rings across eight independent animals, with at least six BRP rings per animal; Kruskal-Wallis test with Dunn’s multiple comparison test, *p*-values were subject to Bonferroni correction). (l) Aversive associative memory performance 3 min after training (short-term memory; STM) markedly reduced in 3d 4xBRP flies in comparison to 3d wild-type 2xBRP flies (*n* = 10–16; Kruskal-Wallis test with Dunn’s multiple comparison test, *p*-values were subject to Bonferroni correction). (m) Aversive associative memory performance at 3 h after training (intermediate-term memory; ITM), anesthesia-resistant memory (ARM), and anesthesia-sensitive memory (ASM) of 3d and 30d 4xBRP flies compared to age-matched control (2xBRP) flies (*n* = 7 independent experiments; Kruskal-Wallis test with Dunn’s multiple comparison test, *p*-values were subject to Bonferroni correction). (n) Aversive olfactory memory performance 3 min after training (STM) higher in appl-gal4 > histone deacetylase-6 (HDAC6) RNAi in comparison to age-matched controls (*n* = 13–21; Kruskal-Wallis test with Dunn’s multiple comparison test, *p*-values were subject to Bonferroni correction). * *p* < 0.05, ** *p* < 0.01, *** *p* < 0.001, ns = not significant, *p* ≥ 0.05. Underlying data is shown in S1 Data.

Presynaptic plasticity processes have been reported to be critical for forming olfactory associative memory in *Drosophila* [11–13]. Based on our findings, we suggest that the scale-up in the size and function of AZ scaffolds is likely to change the “operational range” of synaptic plasticity processes and thus change the threshold for memory formation. Thus, we wanted to test whether genetically provoking an artificial enlargement of AZ scaffolds, independent of the aging process, might affect memory formation. Since BRP is a major essential building block of the AZ scaffold in *Drosophila* [26–28,32], we decided to increase the gene copy number of BRP from two to four copies by combining two additional genomic copies of *brp* [28] with two endogenous copies. As a result, BRP signals increased substantially in 3d flies expressing four-copy BRP (4xBRP) when compared to 3d flies expressing two-copy BRP (2xBRP; Fig 5A–5E). Additionally, RBP levels also increased concomitantly with BRP (Fig 5A–5D and 5F), consistent with the suggested role of BRP to operate as a “master molecule” in shaping the size (and functional performance) of the AZ scaffold [28,29,36]. In order to confirm that the increase in BRP levels resulted in an increase of the average size of AZ scaffolds, we took advantage of STED imaging. Again, a considerable increase in the ring diameter of BRP spots was observed in 2xBRP flies with advancing age (Fig 5G–5K). Meanwhile, we found young flies (3d) expressing 4xBRP to have increased BRP ring diameters when compared to age-matched control flies (2xBRP), and the ring diameter of BRP spots in 4xBRP flies remained rather unchanged with age (Fig 5G–5K).

Having created a genetic state wherein levels of AZ core scaffold proteins increased prematurely in young animals, we decided to investigate the influence of this manipulation on memory formation. Before doing so, however, we wanted to ascertain whether the innate behavior was affected in 4xBRP flies. Thus, we measured naïve odor response and shock reactivity and found 4xBRP flies to show odor avoidance and shock reactivity scores that were indistinguishable from 2xBRP age-matched control flies (2xBRP; S1 Table). Subsequently, we started by measuring short-term memory (STM), and found 4xBRP flies to exhibit lower memory scores “already” at a young age (3d), and their memory scores declined only negligibly with age (Fig 5L). In contrast, control flies (2xBRP) exhibited normal AMI (Fig 5L).

As mentioned earlier, intermediate-term memory (ITM) has also been reported to decline with age [2–4]. Consistently, we found that 30d 2xBRP flies show substantially reduced ITM scores (measured 3-h post-training) when compared to 3-d 2xBRP flies (Fig 5M). By contrast, the 4xBRP flies showed lower ITM scores at a young age (3 d) and, again, the ITM scores did not decrease further in 30-d 4xBRP flies (Fig 5M). In fact, the learning performance of 3-d 4xBRP flies was comparable to that of 30-d 2xBRP flies. Based on distinct genetic mutants and specific pharmacological sensitivities [2,4,38,39], the ITM can be dissected into anesthesia-sensitive memory (ASM) and anesthesia-resistant memory (ARM) components. The ASM, unlike the ARM, has been shown to be strongly impaired with aging [3,4]. The ASM can be calculated by subtracting ARM scores, measured after amnestic cooling, from ITM. Consistent with previous studies [2–4,40], we found ARM in 2xBRP and 4xBRP flies to remain relatively unaffected with age (Fig 5M). In contrast, ASM was nearly absent in 30-d 2xBRP flies when compared to 3-d 2xBRP flies. Reaffirming our idea, we found the young (3-d) 4xBRP flies to show lower ASM scores in comparison to age-matched control (2xBRP) flies, while their ASM scores declined negligibly with age (Fig 5M). These experiments indicate that a genetically provoked “up-scaling” of the average AZ scaffold size is sufficient to induce an “early” decline in memory, similar to AMI, which physiologically occurs over a time course of 20–30 d.

A reduction in BRP levels, per se, might be expected to slow down the onset of AMI. To address this possibility, we removed a single gene copy of *brp*, and found BRP heterozygotes (*brp*^*69*^*/+* or 1xBRP) to exhibit a considerable reduction in the levels of both BRP and RBP (S14A–S14F Fig), indicating that our antibody stainings can detect subtle changes and reaffirming that BRP levels can directly steer the local amounts of other AZ components in the *Drosophila* brain. We found that 3d flies expressing only one BRP copy (*brp*^*69*^*/+*) displayed memory scores comparable to 3d control flies (2xBRP); however, these *brp*^*69*^*/+* flies still exhibited a normally-occurring AMI (30d; S14G Fig). AZ scaffold-dependent control of neuronal plasticity is undoubtedly a complex process [24,41], and other mechanisms, operating in parallel to modulations in the amounts of scaffold proteins, might well contribute to the pace and extent of AMI. Lysine-acetylation of BRP was recently identified as a major node to control the SV release at larval AZs [42,43]. In particular, the loss of histone deacetylase-6 (HDAC6) was found to cause hyperacetylation of BRP and provoke a reduction in evoked SV release at AZs [43]. Interestingly, using immunoprecipitation followed by mass spectroscopic analysis, we found at least 13 lysine sites within BRP to be target for (de)acetylation, (S15 Fig). Next, we asked whether loss of HDAC6 might affect memory. While the odor avoidance and shock reactivity were mainly unaffected by knockdown of *hdac6* (S1 Table), memory scores of both young and aged flies with pan-neuronal knockdown of *hdac6* were higher than those of age-matched driver controls (Fig 5N). These findings are consistent with the idea that driving down the AZs towards the lower limit of their operational range might facilitate memory formation in aged animals. Though any implications of acetylation of BRP or potentially other AZ scaffold proteins with respect to aging process still require extensive analysis, this result shows that BRP-directed modifications, reported to reduce SV release, can in fact increase the efficacy of memory formation in aged animals.

### Homeostasis of Odor-Driven Neuronal Ca^2+^ Signals in Aged Flies

**Fig 6:**
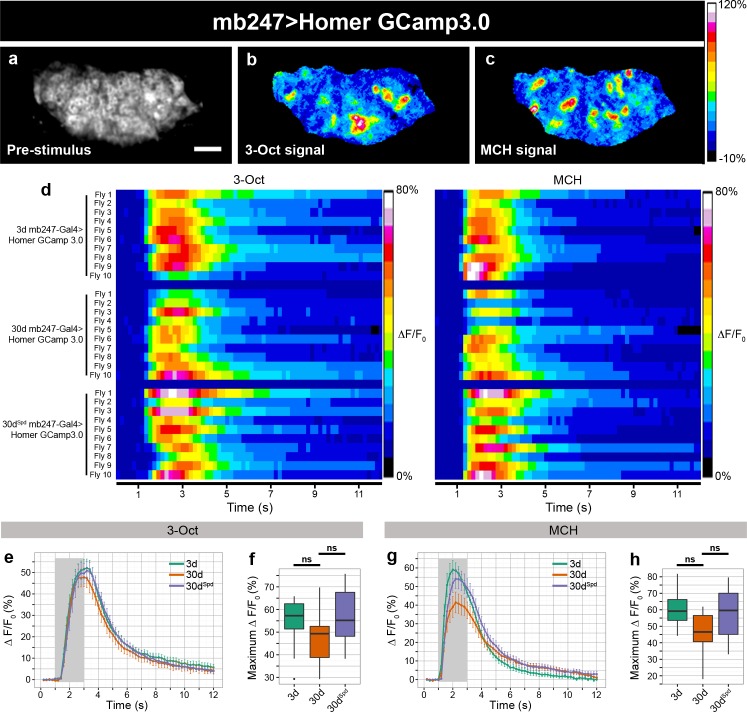
Imaging of Homer-GCamp3.0 within the dendritic claws of KCs to measure odor-evoked Ca^2+^ activity. (a) Expression of Homer-GCamp3.0 in the dendritic claws of KCs and imaged within the calyx region (mb247 > Homer-GCamp3.0). Scale bar: 10 μm. (b–c) False color-coded image of Homer-GCamp3.0 activity within the postsynaptic terminals of KCs in response to 3-Oct and MCH shown in (a). Warm colors indicate high activity and cold colors indicate low or no Ca^2+^ activity. The numbers indicate changes in fluorescence (*ΔF/*F in %). (d) Odor-evoked postsynaptic Ca^2+^ activity, measured by changes in fluorescence of Homer-Gamp3.0, of an individual fly over time, shown as false colors in dendritic claws of KCs in the calyx region. The left panel is in response to the odorant 3-Oct, and the right panel is in response to MCH (*n* = 10 flies). (e) Time course of Ca^2+^ activity induced by 3-Oct in the dendritic terminals of KCs within the calyx region of 3d, 30d, and 30d^Spd^ animals (GCamp3.0 response averaged across three odor exposures from ten flies). (f) Maximum change in GCamp3.0 fluorescence (ΔF*/F* in %) in response to 3-Oct within dendritic claws of KCs of 3d, 30d, and 30d^Spd^ flies (GCamp3.0 response averaged across three odor exposures from ten flies; Kruskal-Wallis test with Dunn’s multiple comparison test, *p*-values were subject to Bonferroni correction). (g) Time course of Ca^2+^ activity induced by MCH in the dendritic terminals of KCs of 3d, 30d, and 30d^Spd^ animals (GCamp3.0 response averaged across three odor exposures from ten flies). (h) Maximum change in GCamp3.0 fluorescence (ΔF*/F* in %) in response to MCH within dendritic claws of KCs of 3d, 30d, and 30d^Spd^ flies (GCamp3.0 response averaged across three odor exposures from ten flies; Kruskal-Wallis test with Dunn’s multiple comparison test, *p*-values were subject to Bonferroni correction). The grey bars indicate the duration of the odor stimuli. * *p* < 0.05, ** *p* < 0.01, ns = not significant, *p* ≥ 0.05. Underlying data is shown in S1 Data.

Finally, we asked how the postsynaptic compartment might respond to these age-associated presynaptic structural and functional changes. To address this question, we used GCaMP3.0 fused to the postsynaptic protein Homer [15] and found the basal expression of Homer-GCamp3.0 to be largely unaffected with age (S2C Fig). Moreover, the sensor was found to be effectively targeted to the postsynaptic density of the PN::KC synapses, as manifested by its specific enrichment within the postsynaptic specializations formed by claw-like dendritic endings of multiple KCs surrounding a single PN bouton (Fig 6A). However, postsynaptic Ca^2+^ signals did not increase with age. Rather, a slight tendency towards a decrease of postsynaptic Ca^2+^ signals was observed in normally aged animals when compared to young controls (Fig 6A–6H). At the same time, aged flies treated with spermidine (30d^Spd^) produced signals more similar to untreated 3d-Homer-GCaMP3.0 flies than to untreated aged animals (Fig 6A–6H). In order to be certain that Homer-GCamp3.0 signals were not saturated, we used high-molar KCl treatment to determine the maximal postsynaptic Ca^2+^ response. Unlike the odor-evoked maximum change in Homer-GCamp3.0 fluorescence of about 55%, KCl stimulation resulted in a substantially higher ΔF/F_0_ value of more than 300% (S16 Fig), suggesting that sensor sensitivity was not a limiting factor for the postsynaptic Ca^2+^ signals. Meanwhile, when the cumulative postsynaptic Ca^2+^ activity was critically analyzed during the odor stimulation, we found that the Ca^2+^ responses reduced significantly in aged (30d) flies relative to young flies, while the Ca^2+^ signals were comparable between young flies and spermidine-fed aged animals (30d^Spd^; S17 Fig).

**Fig 7:**
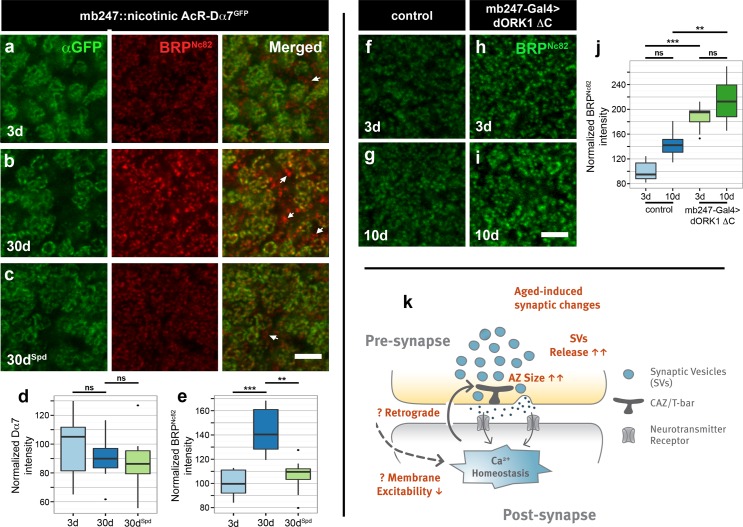
Homeostasis at PN::KC synapses of aged flies. (a–c) Mushroom body calyx of 3d and 30d mb247::Dα7^GFP^ flies and 30d^Spd^ mb247:: Dα7^GFP^ flies immunostained for GFP-labeled Dα7 as well as BRP (corresponding single z-planes are shown). Scale bar: 10 μm. Arrows indicate the recurrent presynapses of KCs that remain unopposed to acetylcholine-receptor rings within calycal neuropil; these KCs presynapses are spatially separated from the sites of cholinergic input onto KCs. (d, e) Quantification of signal intensity of Dα7 (using anti-GFP) and BRP (using Nc82) in the calyx region normalized to 3d flies (*n* = 8–10 independent calyces; Kruskal-Wallis test with Dunn’s multiple comparison test, *p*-values were subject to Bonferroni correction). * *p* < 0.05, ** *p* < 0.01, ns = not significant, *p* ≥ 0.05. (f–i) BRP immunostained within mushroom body calyx from adult brains of 3d and 10d flies expressing UAS-dORK1 ΔC in the KCs compared to age-matched controls. (j) Quantification of signal intensity of BRP (using Nc82) in the calyx region normalized to 3d flies (*n* = 10–12 independent calyces; Kruskal-Wallis test with Dunn’s multiple comparison test, *p*-values were subject to Bonferroni correction). (k) Model showing the age-induced synaptic changes (in red). In the aged brain, the lowering of postsynaptic response with age, due to decrease in membrane excitability or Ca^2+^ homeostasis, might steer retrograde changes in the architecture of AZs. As a result, the AZ characterized by T-bar in flies enlarges in size, leading to higher release of SVs and causing aged synapses to function near the top of their presynaptic plasticity range, leaving little room for additional synaptic strengthening, and possibly impeding further learning. Underlying data is shown in S1 Data.

PNs provide cholinergic input to the KCs within the calyx [44]. We used a fusion of mushroom body-specific enhancer mb247 to the Dα7 subunit of the acetylcholine receptor (mb247::Dα7^GFP^) to explicitly visualize postsynaptic acetylcholine receptors. We showed previously that expression of Dα7-GFP from KCs localized specifically to the KC postsynaptic densities, where it closely matched the AZs of the PNs [45]. While we observed an age-related increase in BRP in 30d mb247::Dα7^GFP^ flies in comparison to 3d mb247::Dα7^GFP^ flies, the levels of Dα7 subunit (quantified using an antibody against GFP fused to the α7 subunit of acetylcholine receptors) did not change with age, and spermidine feeding had no effect on the level of the α7 subunit of acetylcholine receptors (Fig 7A–7E). Similarly, when we stained for endogenous Drep2, a postsynaptic scaffold protein that is known to express strongly within the postsynaptic densities of PN::KC synapses [46], we also found Drep2 to remain unchanged with age (S18 Fig).

At first glance, the increase in release of SVs might be expected to translate into increased postsynaptic responses; however, ample evidence from various studies in different model organisms, including *Drosophila*, support the existence of homeostatic controls, allowing neurons to remain within a certain range of excitation [47,48] in order to avoid epileptic states and Ca^2+^-induced degeneration. In an attempt to directly examine the existence of such homeostatic controls, we wanted to determine whether an increase in the amount of depolarization required to trigger an action potential might influence the architecture of the apposed AZ scaffold. To achieve this, we used dORK1**Δ**C, a constitutively open K^+^ selective pore that causes hyperpolarization of neurons and subsequent inactivation of neuronal function [45,49]. dORK1**Δ**C was specifically expressed in the KCs, and presynaptic terminals of PNs within the calyx were analyzed for BRP levels (Fig 7F–7I). Indeed, we found a substantial increase in the levels of BRP in the calyces of both 3d as well as 10d mb247>dORK1**Δ**C flies, when compared to age-matched controls (Fig 7J). Thus, a drop in postsynaptic excitability can drive a homeostatic increase in presynaptic AZ scaffolds, leading to a potential increase in SV release at olfactory synapses—a finding similar to the one we found at aging synapses. Though the exact mechanisms allowing for homeostatic compensation of the elevated presynaptic release remain to be further worked out, it is tempting to speculate that homeostatic mechanisms coupling postsynaptic excitability to presynaptic release function might drive aging synapses towards the upper limit of their operational range and be critically involved in AMI (see model in Fig 7K)

## Discussion

The aging process, causing progressive deterioration of an organism, is subject to a complex interplay of regulatory mechanisms. One of the primary aims of aging research is to use the understanding of this process to delay or prevent age-related pathologies, including AMI. We previously showed that restoration of polyamine levels by dietary supplementation with spermidine suppressed AMI in fruit flies [3], providing us with a protective paradigm to identify candidate processes that might be functionally associated with AMI. As an insight towards the synaptic basis of AMI, we describe an age-induced increase in the levels of core AZ proteins, BRP, and RBP and of the functionally critical release factor Unc13, together with a shift towards an enlargement of AZ scaffolds within the olfactory system. In addition, based on SynpH experiments, we observed a substantial increase in the release of SVs at aged synapses (PN-to-KC and KC-to-mushroom body output neuron [MBON] synapses) in response to odors used for learning experiments. Importantly, spermidine feeding was able to “protect” from both the functional and structural changes at aged AZs, arguing in favor of specific synaptic changes to be causally relevant for AMI. Indeed, installing 4xBRP not only increased the size of BRP rings in young flies, similar to those found in aged animals, but also provoked memory impairment in young flies.

Notably, a reduction of BRP levels has previously been reported to affect ARM but not ASM [50]. Here, we report that an increase in BRP levels (by changing the gene copy number of BRP from two to four copies) severely affected ASM. These findings suggest that the two complementary forms of memory (ARM and ASM) might rely on the recruitment of distinct presynaptic “functional modules.” The loss of *brp* has been shown to severely reduce release function in response to single low frequency, but not in response to high-frequency stimulation [27], indicating that SV release at low-frequency stimulation might be particularly relevant for forming ARM, a memory component that develops gradually after training. On the other hand, mobilization of the SVs during high frequency stimulation has been suggested to be critical for formation of ASM [50], a memory component that predominates early memory and decays with age. Thus, the increase in the size of the AZ scaffolds might potentially contribute directly to AMI by interfering with mechanisms facilitating SV availability in the course of forming ASM.

Though the exact mechanisms underlying age-induced synaptic changes remain to be fully worked out, a reduction in autophagy-mediated protein degradation might well be involved [51–53]. Autophagy is a cellular digestion pathway that involves the sequestration of cytoplasmic components within a double-membrane vesicle called autophagosome, which fuses with lysosomes (autolysosomes) to degrade autophagic cargo by acidic hydrolases [52]. Interestingly, spermidine was shown to induce autophagy in several model systems, including rodent tissues and cultured human cells [51,54,55]. Moreover, amelioration of a-synuclein neurotoxicity due to spermidine administration was accompanied by autophagy induction [56]. Of note, we also found that spermidine feeding prevented accumulation of poly-ubiquitinated proteins by plausibly halting normally occurring age-induced decline of autophagic clearance [3,57]. The gene *atg7* encodes an E1-like enzyme required for activation of both Atg8 and Atg12, a step critical for the completion of the autophagic pathway [53]. We found that *atg7*-mutant flies (*atg7*^-/-^) exhibit reduced memory scores at a young age (3d), which declined further with age (20-d of age or 20d) [3]. Concurrently, spermidine-mediated protection from memory impairment was eliminated in *atg7*^−/−^ flies (for both 3d- and 20d-flies) [3,57]. Therefore, we wondered whether the decrease in the autophagic pathway might, per se, provoke increase of AZ scaffold components. When staining for BRP in *atg7*-mutant brains (*atg7*^*-/-*^), we found a brain-wide increase in levels of BRP (for both BRP^Nc82^ and BRP^N-term^ antibodies), and spermidine feeding was unable to prevent this age-related increase (S19 Fig). The finding that spermidine feeding in *atg7*^−/−^ flies neither blocked the increase in BRP levels (S19 Fig) nor suppressed memory impairment [3] suggests that the integrity of the autophagic system is crucial for the spermidine-mediated protection from age-associated increase in AZ scaffold components. Spermidine effects were recently shown to involve widespread changes of both nuclear and cytosolic protein acetylation [58,59].

In primary neurons, autophagosomes have previously been observed to form at the distal end of the axon, indicating compartmentalization and spatial regulation of autophagosome biogenesis [60,61]. More recently, autophagosomes were demonstrated to form directly near synapses and were found to be required for presynaptic assembly at developing synaptic terminals of *Caenorhabditis elegans* [62]. Moreover, the crucial release factor Unc13 was found to accumulate under conditions of defective endosomal microautophagy (a specialized form of autophagy) at developing neuromuscular synapses of *Drosophila*, suggesting Unc13 to be a substrate of this form of autophagy [63]. Interestingly, we have shown recently that the synaptic levels of Unc13-A isoform scale tightly with the levels of the BRP/RBP scaffold [64]. Thus, it is conceivable that some of the AZ proteins, whose levels increase with age (BRP/RBP/Unc13), might be direct substrates of “pre-synaptic autophagy,” and that spermidine feeding might augment effective autophagic degradation of these proteins at aging synapses.

We also observed a moderate decrease in synapse numbers in aging *Drosophila* brains, a phenotype that was unaffected by spermidine feeding. Our data compare favorably with studies in mammals. For example, loss of synapses in aged rodents has been reported in the dentate gyrus as well as the CA1 area of the brain [8,65,66]. Additionally, the “unitary” intracellular-evoked amplitude elicited by minimal stimulation protocols has been found to be greater in old than in young rodents [67], suggesting that the “surviving synapses” are stronger [68]. It is of note that the induction threshold for long-term potentiation, considered to be a synaptic correlate of learning, has been reported to increase in aged rodents [10]. Similarly, an age-related increase in the amplitude of endplate potentials evoked has been reported at mouse neuromuscular synapses [69,70]. By contrast, a study at neuromuscular junctions of *C*. *elegans* revealed that aged motor neurons undergo a progressive reduction in synaptic transmission [71]. In flies, however, an age-related increase in the amplitude of the excitatory postsynaptic potential at adult neuromuscular junctions has been reported recently; this increase was suggested to tune the response of the homeostatic signaling system and establish a new homeostatic set point [72]. Collectively, these findings suggest that the dynamic range of synaptic plasticity may change with advancing age and, thus, contribute to AMI.

Why would an increase in the odor-evoked SV release and ultrastructural size of AZ scaffolds impair the efficacy of forming new memories? Synapses appear to display a “finite ceiling and floor” that define a synaptic operating range [73]. In rodents, the formation of new memories seems to drive synaptic strength to the upper limit of a fixed operating range, thereby creating an imbalance [73]. As a result, if the synapses are not returned to the midpoint of the synaptic modification range, then additional strengthening required for new memory formation might be blocked, and the system is driven to employ homeostatic compensatory mechanisms to balance the change [74]. In our experiments, we found dendritic Ca^2+^ signals and postsynaptic receptor levels to remain largely unchanged with age, suggesting the existence of homeostatic mechanisms that might allow the up-scaling of presynaptic release to be compensated by lowering the postsynaptic response to a given amount of neurotransmitter released. On the other hand, this upscaling of presynaptic structure and function might also be a homeostatic response to a reduction in postsynaptic excitability or Ca^2+^ homeostasis, steering retrograde enlargement of AZ scaffold and higher release of SVs (Fig 7K). In fact, the influx of postsynaptic Ca^2+^ through glutamate receptors at the peripheral glutamatergic synapses of *Drosophila* has been reported to control presynaptic assembly by retrograde signalling [47,48,75]. While the exact nature of homeostatic controls connecting pre- with postsynaptic neurons in the olfactory system remains to be resolved, changes in plasma membrane excitability, a change in postsynaptic neurotransmitter sensitivity, or an increase in inhibitory GABAergic drive are obvious candidate processes. Taken together, we propose these synaptic changes steer the presynaptic AZs to function towards the upper limit of their operational range, making these synapses unable to react adequately to conditioning stimuli and provoke potentiation or depression of synapses in order to encode memory formation [11,12,76].

Sleep is widely believed to be critical for formation and consolidation of memories [77]. In sleep-deprived animals, neuronal circuits would exceed available space and/or saturate, thereby affecting an individual’s ability to learn [77]. Importantly, sleep deprivation has also been associated with widespread increases of BRP levels in the *Drosophila* brain [78]. Notably, we also observed a brain-wide increase in BRP levels in aged brains. It is tempting to speculate that both sleep deprivation and aging change the operational range over several synaptic relays and thereby affect memory formation—a topic that deserves further investigation in future.

Taken together, our data show that upscaling of presynaptic structure and function contribute to an AMI in *Drosophila*. Furthermore, and restoration of polyamine levels prevents these age-associated alterations as well as AMI. Thus, spermidine feeding provides a unique opportunity to further the molecular and functional dissection of the mechanisms underlying AMI with the ultimate goal of restoring memory function in older humans.

## Materials and Methods

### Animal Rearing and Fly Strains

All fly strains were reared under standard laboratory conditions [79] at 25°C and ≈70% humidity, with constant 12:12 h light/dark cycle. Flies from an isogenized *w*^1118^ strain were used as the wild-type control for all experiments. Flies carrying P(acman) *cac*^*GFP*^, P(acman) *brp83*^*GFP*^ and P(acman) *brp83* [28] and mb247::Dα7^GFP^ [45] were described previously. The generation of UAS-homer-GCaMP3.0 flies are described elsewhere [15]. Briefly, cDNA of *dhomer* was amplified from *w* ^*1118*^ flies and inserted with a C-terminal linked GCaMP3^36^ into pUAST. Both UAS-GCamp3.0 (on the 3rd chromosome) [80] and UAS-SynpH [81] were kindly provided by Gero Miesenböck. *Atg7*^*d14*^ and *Atg7*^*d77*^ flies were kind gifts from Thomas Neufeld [53]. In addition, mb247-Gal4 [82] and gh146-Gal4 [83] were used.

As previously described [3], the fly food was prepared according to Bloomington media recipe (www.flystocks.bio.indiana.edu/Fly_Work/media-recipes/media-recipes.htm) with minor modification, which was called Spd^−^or normal food. Spermidine (Sigma Aldrich) was prepared as a 2 M stock solution in sterile distilled water, aliquoted in single-use portions and stored at −20°C. After food had cooled down to 40°C, Spermidine was added to normal food to a final concentration of 1 mM or 5 mM Spd, and called Spd^1mM+^ or Spd^5mM+^, respectively. Parental flies mated on either Spd^−^or Spd^5mM+^ food for all experiments, and their progeny were allowed to develop on the respective food. Flies used in all experiments were F1 progeny. The flies were collected once a day for aging, as a results-specific age indicated is day ± 24 h.

### Behavioral Assays

Behavioral experiments were performed in dim red light at 25°C and 80% relative humidity with 3-Oct (1:150 dilution in mineral oil presented in a 14 mm cup) and MCH (1:100 dilution in mineral oil presented in a 14 mm cup) serving as olfactory cues, and 120V AC current serving as a behavioral reinforcer. Standard single-cycle olfactory associative memory was performed as previously described [3,4,46,84,85], with minor modifications. Briefly, about 60–80 flies received one training session, during which they were exposed sequentially to one odor (conditioned stimulus, CS^+^; 3-Oct or MCH) paired with electric shock (unconditioned stimulus, US) and then to a second odor (CS^−^; MCH or 3-Oct) without US for 60 s with 30 s rest interval between each odor presentation. During testing, the flies were exposed simultaneously to the CS^+^ and CS^−^ in a T-maze for 30 s.

The conditioned odor avoidance was tested immediately after training for STM (memory tested immediately after odor conditioning). Subsequently, flies were trapped in either T-maze arm, anesthetized, and counted. From this distribution, a performance index was calculated as the number of flies avoiding the shocked odor minus the number avoiding the nonshocked odor divided by the total number of flies and, finally, timed by 100. A 50:50 distribution (no learning) yielded a PI of zero, and a 0:100 distribution away from the CS^+^ yielded a PI of 100. A final performance index was calculated by the average of both reciprocal indices for the two odors.

For ITM, flies were trained as described above, but tested 3 h after training. As a component of ITM, ARM was separated from ASM by cold-amnestic treatment, during which the trained flies were anesthetized 90 s on ice at 30 min before testing. In the end, ASM was calculated by subtracting the performance index of ARM from that of ITM for each training session on the same day, respectively.

### Staining Protocol

#### Whole-mount adult brains

Adult brains were dissected in HL3 (which contains 70 mM NaCl, 5 mM KCl, 20 mM MgCl_2_, 10 mM NaHCO_3_, 5 mM Trehalose, 115 mM Sucrose, 5 mM Hepes, added to 500 ml H_2_O; pH adjusted to 7.2) on ice and immediately fixed in cold 4% Paraformaldehyde (v/v) for 20 min at 20–30°C. After fixation, the brains were incubated in 1% PBT (phosphate-buffered saline (PBS) containing 1% Triton X-100; v/v) for 20 min and then preincubated in 0.3% PBT (PBS containing 0.3% Triton X-100) with 10% normal goat serum (NGS; v/v) at 20–30°C. For primary antibody treatment, samples were incubated in 0.3% PBT containing 5% NGS and the primary antibodies for 48 h at 20–30°C. After primary antibody incubation, brains were washed in 0.3% PBT, four times for 30 min at 20–30°C, and then overnight at 4°C. All samples were then incubated in 0.3% PBT with 5% NGS containing the secondary antibodies for 24 h at 20–30°C. Brains were washed again four times for 30 min at 20–30°C, then overnight at 4°C. Brains were finally mounted in Vectashield overnight before confocal scanning (Vector Laboratories).

#### Image acquisition, processing, and analysis

The images were acquired, processed, and analyzed as previously described [46,85], with minor modifications. Briefly, conventional confocal images were acquired at room temperature with a Leica Microsystems TCS SP8 confocal microscope using a 63x 1.4 NA oil objective for detailed scans and a 20x 0.7 NA oil objective for overview scans. All images were acquired using Leica LCS AF software. Lateral pixel size was approximately 300 nm for overview scans, approximately 100 nm for detailed scans. Typically, 1,024 x 1,024 images were scanned at 400 Hz using 4x line averaging. Images of calyces for cryostat sections were acquired at room temperature with a Leica Microsystems TCS SP5 CW STED microscope in confocal mode using a 100x 1.4 NA oil objective. STED images were acquired on a Leica TCS STED CW. Images were deconvolved using the built-in deconvolution algorithms of the Leica LAS-AF software. The PSF was generated by using a 2-D Lorentz function with the full-width half-maximum set to 60 nm (as calculated on the image using the Wiener filter algorithm; regulation parameter: 0.05).

In order to analyze the brain scans, the signal intensity within a neuropil of interest (whole central brain, or CB; antennal lobes, or AL; and calyx) was determined using Amira software (Amira 5.3.3, FEI Visualization Sciences Group, Oregon, US). The region of interest within the 3-D image stack was masked using the tool Segmentation Editor by interpolating manual selections between slices. Average intensity values were calculated for all pixels within each mask for each channel separately.

For STED analysis, deconvolved BRP spots (stained with monoclonal Nc82 antiboby) were processed in ImageJ. The diameters of planar oriented BRP rings were measured using the line tool of ImageJ. The distance from intensity maximum to intensity maximum was acquired in the plot window of individual hand-drawn lines and transferred to Microsoft Excel.

### EM

#### Conventional EM

Brains were dissected in HL3 solution and fixed for 20 min at room temperature with 4% paraformaldehyde and 0.5% glutaraldehyde in a PBS. Subsequently, the brains were incubated overnight at 4°C with 2% glutaraldehyde in buffer containing 0.1 M sodium cacodylate at pH 7.2. Brains were then washed three times in cacodylate buffer for 10 min at 20–30°C. Afterwards, the brains were incubated with 1% Osmium tetroxide and 0.8% KFeCn (in 0.1 M cacodylate buffer) for 90 min on ice. Brains were then washed with cacodylate buffer for 10 min on ice and then three quick washes with distilled water. The brain were stained with 1% Uranylacetate (w/v) for 90 min on ice and dehydrated through a series of increasing alcohol concentrations. Samples were embedded in EPON resin by incubation sequentially in ethanol/EPON 1:1 solution for 45 min and 90 min at 20–30°C, then in pure EPON overnight at 15–20°C. Thereafter, the resin was changed once and brains were embedded in a single block at 60°C to allow for polymerization of the resin.

Following embedding, sections of 60 nm, each, were cut using a Leica Ultracut E ultramicrotome equipped with a 2 mm diamond knife. Sections were collected on 100 mesh copper grids (Plano GmbH, Germany) coated with 0.1% Pioloform resin. Contrast was enhanced by placing the grids in 2% uranyl acetate for 30 min, followed by washing with water three times and, then, incubation in lead citrate for 2 min. The grids were washed three times with water and dried. Images were acquired fully automatically on a FEI Tecnai Spirit transmission electron microscope operated at 120 kV equipped with a FEI 2K Eagle CCD camera using Leginon [86]. Regions of interest were first selected at 560x nominal magnification and then successively imaged at 4,400x and 26,000x nominal magnification, respectively. Series of more than 1,000 TEM images were then stitched to a single montage covering nearly the full calyx region using the TrakEM2 software [87] implemented in Fiji [88].

### Immuno-EM

Brains were dissected in HL3 solution and fixed for 20 min at room temperature with 4% paraformaldehyde and 0.2% Glutaraldehyde in a buffer containing 50 mM Sodium Cacodylate and 50 mM NaCl at pH 7.5. Afterwards, brains were washed twice in the buffer and dehydrated through a series of increasing alcohol concentrations. Samples were embedded in London-Resin (LR)-Gold resin by incubating them in Ethanol/LR-Gold 1:1 solution overnight at 4°C, followed by Ethanol/LR-Gold 1:5 solution for 4 h at room temperature. Thereafter, the samples were washed first with LR-Gold/0.2% Benzil overnight, a second time for 4 h, and then again overnight. Finally, the brains were placed in BEEM capsules covered with LR-Gold/0.2% Benzil resin and placed under a UV lamp at 4°C for 5 d to allow for polymerization of the resin.

Following embedding, sections 70–80 nm, each, were cut using a Leica Ultracut E ultramicrotome equipped with a 2 mm diamond knife. Sections were collected on 100 mesh nickel grids (Plano GmbH, Germany) coated with 0.1% Pioloform resin and transferred to a buffer solution (20 mM Tris-HCl, 0.9% NaCl, pH 8.0). Prior to staining, sections were blocked for 10 min with 0.04% BSA in buffer. Sections were incubated with the primary antibody (guinea pig-anti RBP^SH3II+III^ and rabbit-anti BRP^last200^, 1:500 dilution) in blocking solution overnight at 4°C. After washing four times in buffer, the sections were incubated in buffer containing the secondary antibody (goat anti-guinea pig 10 nm colloidal gold, goat anti-rabbit 5 nm colloidal gold British Biocell, 1:100) for 2–3 h at room temperature. Finally, the sections were washed four times in buffer and three times in distilled water. Contrast was enhanced by placing the grids in 2% uranyl acetate for 30 min, followed by washing three times with water and, afterwards, incubation in lead citrate for 2 min. Afterwards, the grids were washed three times with water and dried. Images were acquired on a FEI Tecnai Spirit, 120 kV transmission electron microscope equipped with a FEI 2K Eagle CCD camera.

### Antibodies Used

The following primary antibodies were used: MαBRP^Nc82^ (ref. 9, 10; 1:100), GPαBRP^N-term^ (1:800) [25,27], RbαRBP^C-term^ (1:800) [29], MαSynapsin (1:20) [89], RatαSyb (1:100) [90], RbαSynaptotagmin-1^C-term^ (1:500) [91], RbαGFP (Molecular Probes; 1:500), RbαDrep2^C-term^ (1:500) [46], RbαUnc13^C-term^ (1:500) [63], RbαBRP^last200^ (1:500), and GPαRBP^SH3II+III^ (1:500).

The following secondary antibodies were used: GαM Alexa 488 (Molecular Probes; 1:400), GαR Alexa 488 (Molecular Probes; 1:500), GαGP Alexa 555 (Invitrogen; 1:800), GαM Cy3 (Dianova; 1:500), and GαR Cy5 (Invitrogen; 1:400).

For Immunoprecipitation, BRP^last200^ and IgG were used at final amount of 50 ug per 500 ul. For western blots, secondary antibody was used at a dilution 1:1,000.

### Optophysiological Imaging of GCaMP3.0, Homer-GCamp3.0 and SynaptopHlourin (SynpH)

Female 3d or 30d flies were briefly anesthetized on ice and immobilized in a small chamber under thin sticky tape. A small window was cut through the sticky tape and the cuticle of the head capsule using a splint of a razor blade. Trachea were carefully removed and the brain was covered with Ringer’s solution (5 mM HEPES, 130 mM NaCl, 5 mM KCl, 2 mM MgCl_2_, 2 mM CaCl_2_, pH = 7.3). Imaging was performed using an LSM 7 MP two-photon microscope (Carl Zeiss) equipped with a mode-locked Ti-sapphire Chameleon Vision II laser (Coherent), a 500–550 nm bandpass filter, and a Plan-Apochromat 20×1.0 NA water-immersion objective (Carl Zeiss). A custom-built device to supply odorous air with a constant flow rate of 1 ml/s directly to the fly’s antennae was attached to the microscope. Odor stimulation (MCH or 3-Oct, diluted 1:100 or 1:150, respectively, in mineral oil or pure mineral oil) was controlled using a custom-written LABVIEW program (National instruments). GCamp3.0, homer-GCaMP, and SynpH were excited at 920 nm and fluorescence monitored at an image acquisition rate of 5 Hz. The odorants were presented with a 20 s break between stimulation, and each fly was exposed to five to six repetitive experiments.

The images were aligned to reduce small shifts in the X–Y direction using a custom written ImageJ plugin. The mean intensity within the region of interest of five images before stimulus onset was used as baseline fluorescence (F_0_). The difference in intensity (ΔF) was calculated by subtracting F_0_ from the fluorescence intensity value within the ROI of each image (F_i_) and, subsequently, divided by the baseline fluorescence. ΔF/F_0_ values of three or more repetitions were averaged for each fly.

Odor-induced fluorescence changes of SynpH were considered in calycal PN boutons showing ΔF/F_0_ values more than twice the standard deviation of the baseline fluorescence. The boutons with the five highest odor-induced ΔF/F_0_ amplitudes were considered for further analysis. We found SynpH to exhibit rapid photo-bleaching, therefore, bleaching correction was performed on its ΔF/F_0_ values. For this, first, ΔF/F_0_ values from the onset of the stimulus until the decay of the signal were removed and then the best least square fit was obtained using the remaining ΔF/F_0_ values (second order polynomial decay function). Subsequently, this decay function was subtracted from the entire original ΔF/F_0_ curve, and the new modified data are the bleaching corrected data.

Fluorescence emission of cytosolic GCamp was determined within specific boutons in the calyx that respond to the odor stimulus, and only the boutons showing ΔF/F_0_ values of more than 100% in four to five stimulations were averaged for each fly and considered for final analysis. Fluorescence changes of mb247-Gal4; UAS-homer-GCamp flies were averaged over the five most responsive microglomerular structures, as anatomically defined by basal fluorescence.

False color-coded images were obtained by subtracting the image just before stimulus onset from the image at the maximum of the intensity difference (i.e., at 2 s after odor onset) and divided by the baseline fluorescence.

The KCl experiments were performed using a fluorescence microscope (Zeiss) equipped with a xenon lamp (Lambda DG-4, Sutter Instrument), a 14-bit CCD camera (Coolsnap HQ, Photometrics) and a 20 × NA = 1 water-immersion objective. Images were acquired at 5 Hz using Metafluor (Visitron Systems). After recording some initial frames, KCl was added to the Ringer’s solution covering the fly brain (final concentration 0.05 M). Fluorescence changes were determined in a circular region covering the calyx (d = 20 μm), and background fluorescence determined outside the calyx was subtracted.

### *D*. *Melanogaster* Head Extract and Immunoprecipitation

For the identification of (de)acetylated residues of BRP, we did “conventional” protein extractions from *Drosophila* heads combined with BRP immunoprecipitations. The protocol could be divided into four main sections. 1) Precoupling of antibodies to matrix (50 ug antibody per reaction): 3 LoBind cups (2 ml; Eppendorf) containing Affiprep Protein A matrix were prepared: 1 X 30ul for specific antibody, 1 X 30ul for IgG control, 1 X 60 ul for head extract preclearing. The cups were washed 3 X with 500 ul H-buffer (25 mM HEPES pH 8.3 (NaOH), 150 mM NaCl, 1 mM MgCl_2_, 1 mM EGTA, 10% Glycerol) by inverting several times, followed by centrifugation 1,000 g_max_ (3,000 rpm) for 1 min. 500 ul H-buffer (+ BRP^last200^ or IgG) per coupling was prepared. 500 ul antibody solution (= 50 ug IgG) was added per 30 ul washed Protein A-beads. Beads were incubated with antibody solution for 2 h on the wheel at 4°C. The Affiprep beads-antibody were collected by centrifugation for 3 min at 1,000 g_max_. Affiprep beads-Antibody were washed 3 X by inverting tubes and 3 X for 10 min on wheel with IP buffer. 2) Homogenizing fly heads from stored fly heads [–80°C]. Fly heads were transferred with a clean spatula into 1 ml glass homogenizer. For 300 ul frozen fly heads, 300 ul Homogenization buffer (without detergent) was added, and heads were sheared at 900 rpm using an electronic overhead stirrer. Samples were collected in LoBind cups (2 ml; Eppendorf). 2 X 300 ul was added to rinse pestle and homogenizer (Total volume in cups ~1,100–1,200 ul). Sodium-deoxycholic Acid (DOC) was added to a final concentration of 0.4% (28 ul of 10% stock spiked into homogenate (1:25 v/v)). Triton X-100 was added to a final concentration of 1% (35 ul of 20% stock spiked into homogenate (1:20 v/v)). The samples (Homogenate) were incubated for 60 min at 4°C at level 8 (slow) on wheel. 20 ul of homogenate was stored for SDS-PAGE analysis for monitoring antigen during extraction/pull-down procedure. Homogenate (H) was centrifuged for 15 min at 17,000 g_max_. Supernatant (yellow in color) was transferred to a fresh LoBind cup. Centrifugation of S1 was repeated 4X to get rid of fat and remaining head debris. After final centrifugation step, remaining supernatant was diluted 1:1 with H-buffer (without detergent). Total volume of Input was ~1,400 ul and of following composition: 25 mM Hepes pH 8.05 (NaOH), 150 mM NaCl, 0.5 mM MgCl_2_, 0.5 mM EGTA, 5% Glycerol, 0.2% DOC, 0,55% Triton X-100. 3) Preclearing of fly head extract on Protein A-IgG beads: Diluted fly head extract was applied to preclearing beads and incubated for 60 min at 4°C while rotating on wheel. Precleared extract was separated by centrifugation for 3 min at 1,000 g_max_. Supernatant (IP input) was recovered. 4) Precipitation: Precleared extract (IP input) was applied to antibody-bead matrix (600 ul to specific Antibody-beads, 600 ul to control IgGs) and antibody–antigen binding was performed overnight at 4°C. Immunoprecipitates were collected by centrifugation at 1,000 g_max_ for 4 min at 4°C. Affiprep Beads-Antibody-Antigen were washed 3 X with a quick rinse followed by 2 X 20 min with 1 mL IP Buffer (H-buffer + 0.5% Triton-X 100 + 0.2% Na-DOC). Affiprep Beads-Antibody-Antigen were resuspended in 1,000 ul IP buffer and transferred to a clean LoBind cup (2 ml; Eppendorf). Affiprep Beads-Antibody-Antigen were centrifuged, and most of the supernatant was removed (without removing beads). 4.) Elution: For elution, 100 ul of 2X Laemmeli Buffer was added to Affiprep Beads-Antibody-Antigen and heated for 10 min at 95°C, 600 rpm, followed by centrifugation for 5 min at 1,000 g_max_. Supernatant (IP eluate) was transferred into a fresh LoBind Cup (2 ml; Eppendorf). Immunoprecipitation was verified with western blot.

### Sample Preparation and Mass Spectrometry

For identification of (de)acetylated lysine residues in BRP, IP eluate was heated in SDS-PAGE loading buffer, reduced with 1 mM DTT (Sigma‐Aldrich) for 5 min at 95°C and alkylated using 5.5 mM iodoacetamide (Sigma‐Aldrich) for 30 min at 20°C. The protein mixtures were separated on 4%–12% gradient SDS‐PAGE (NuPAGE, Invitrogen). The gel lanes were cut into ten equal slices, the proteins were in-gel digested with trypsin (Promega) [92], and the resulting peptide mixtures were processed on STAGE tips [93] and analyzed by LC-MS/MS.

Mass spectrometric (MS) measurements were performed on an LTQ Orbitrap XL mass spectrometer (Thermo Fisher Scientific) coupled to an Agilent 1200 nanoflow–HPLC (Agilent Technologies GmbH, Waldbronn, Germany) [94]. HPLC–column tips (fused silica) with 75 μm inner diameter (New Objective, Woburn, MA, USA) were self-packed with Reprosil–Pur 120 ODS–3 (Dr. Maisch, Ammerbuch, Germany) to a length of 20 cm. Samples were applied directly onto the column without a precolumn. A gradient of A (0.5% acetic acid (high purity, LGC Promochem, Wesel, Germany) in water and B (0.5% acetic acid in 80% acetonitrile (LC–MS grade, Wako, Germany) in water) with increasing organic proportion was used for peptide separation (loading of sample with 2% B; separation ramp: from 10%–30% B within 80 min). The flow rate was 250 nl/min and for sample application 500 nl/min. The mass spectrometer was operated in the data-dependent mode and switched automatically between MS (maximum of 1 x 10^6^ ions) and MS/MS. Each MS scan was followed by a maximum of five MS/MS scans in the linear ion trap using normalized collision energy of 35% and a target value of 5,000. Parent ions with a charge state from z = 1 and unassigned charge states were excluded for fragmentation. The mass range for MS was m/z = 370–2,000. The resolution was set to 60,000. MS parameters were as follows: spray voltage 2.3 kV; no sheath and auxiliary gas flow; ion transfer tube temperature 125°C.

#### Identification of proteins and protein ratio assignment using MaxQuant

The MS raw data files were uploaded into the MaxQuant software version 1.4.1.2 [95] for peak detection, generation of peak lists of mass error corrected peptides, and for database searches. A full-length UniProt *D*. *melanogaster* database additionally containing common contaminants such as keratins and enzymes used for in-gel digestion (based on UniProt *Drosophila* FASTA version December 2013) was used as reference. Carbamidomethylcysteine was set as fixed modification, methionine oxidation, protein amino-terminal acetylation, and lysine acetylation were set as variable modifications, and label-free was chosen as quantitation mode. Three missed cleavages were allowed, enzyme specificity was trypsin/P, and the MS/MS tolerance was set to 0.5 Da. The average mass precision of identified peptides was in general less than 1 ppm after recalibration. Peptide lists were further used by MaxQuant to identify and relatively quantify proteins using the following parameters: peptide and protein false discovery rates, based on a forward-reverse database, were set to 0.01, minimum peptide length was set to seven, minimum number of peptides for identification and quantitation of proteins was set to two, of which one must be unique, minimum ratio count was set to two, and identified proteins were requantified. The “match-between-run” option (2 min) was used.

To analyze acetylation status of BRP, the data was processed using the freely available Perseus software (Cox et al, 2011). For each IP, average acetylation intensity was calculated out of intensities of all sites identified in each replicate normalized to the respective protein intensity of BRP.

### Statistics

Data were analyzed in R v3.1.2 using the additional CRAN package dunn.test v1.2.2. Asterisks are used in the figures to denote significance: * *p* < 0.05, ** *p* < 0.01, *** *p* < 0.001, ns = not significant. Nonparametric methods were used because of the small sample sizes and because of failure of tests for normality for parts of the data (Shapiro-Wilk test). Unless indicated otherwise, the different groups in each figure were first compared using the Kruskal-Wallis test, followed by Dunn’s test for posthoc multiple comparisons. Nonparametric tests were used in order to avoid being biased by outliers, which are represented by solid circles. All *p*-values that are reported have been subject to Bonferroni correction for the number of comparisons. Additional relevant information is indicated in the figure legends. The data for the behavioral studies were collected with the investigator blind to the genotypes, treatment, and age of genotypes. There was no blinding in the other experiments. The data were collected and processed side by side in randomized order for all experiments. In order to analyze the difference in Homer-GCaMP3.0 responses (Fig 6 and S17 Fig), two-sided Kolmogorov-Smirnov tests were conducted in R, and the GCaMP3 responses only during odor stimulation and were compared.

## Supporting Information

S1 Data Excel spreadsheet containing, in separate sheets, numerical data underlying panels 1e–1h, 2d, 2h, 2l–2n, 3e–3g, 4d, 4h, 4m–4n, 5e–5f, 5k, 5m–5n, 6e–6h, 7d–7e, 7j, S1, S2a–S2c, S3e–S3h, S4a–S4f, S5b–S5c, S6a–S6d, S7d, S8e–S8g, S9d, S10d–S10e, S11d–S11f, S14e–S14g, S16b–S16c, S17, S18d–S18f, S19e–S19f, S1 Table. (XLSX)

S1 Fig Aversive olfactory memory as a function of stimulation intensity in aged flies. STM index plotted against shock number as experienced during training sessions with 120 V DC in 3d (light blue bars) and 30d (dark blue bars) wild-type *w*^*1118*^ flies (*n* = 6–8; Kruskal-Wallis test with Dunn’s multiple comparison test, *p*-values were subject to Bonferroni correction). * *p* < 0.05, ** *p* < 0.01, *** *p* < 0.001, ns = not significant, *p* ≥ 0.05. Underlying data is shown in S1 Data.
(TIF)

S2 Fig Basal fluorescence of different sensors (within the calyx neuropil) used for optogenetic analysis. (a) Quantification of levels of GCamp3.0 in the PN terminals within the calyx region normalized to 3d flies (*n* = 6–7 independent calyces; Kruskal-Wallis test). (b) Quantification of levels of SynaptopHlourin (SynpH) in the PN terminals within the calyx region normalized to 3d flies (*n* = 7–12 independent calyces; Kruskal-Wallis test). (c) Quantification of levels of Homer-GCamp3.0 in the dendritic claws of KCs within the calyx region normalized to 3d flies (*n* = 10–12 independent calyces; Kruskal-Wallis test). ns = not significant, *p* ≥ 0.05. Underlying data is shown in S1 Data.
(TIF)

S3 Fig Ca^2+^-imaging in the PNs within the calyx region in response to odors in aged flies. (a) Expression of GCaMP3.0 in the PNs and imaged within the calyx neuropil. (b, c) False color-coded image of Ca^2+^ activity within the presynaptic terminals of PNs in response to 3-Oct and MCH shown in (a). Warm colors indicate high levels, while cold colors low levels or no Ca^2+^ activity. The numbers indicate changes in fluorescence (ΔF*/F* in %). Scale bar: 10 μm. (d) Odor-evoked Ca^2+^ activity, measured by changes in fluorescence of Gamp3.0, of an individual fly over time, shown as false colors in the presynaptic terminal of PNs in calyx region, in response to the odorants 3-Oct and MCH. (GCamp3.0 response averaged across three odor exposures from 6–7 animals). (e) Time course of Ca^2+^ activity induced by 3-Oct (averaged across three odor exposure) in the presynaptic terminals of PNs within calyx neuropil of 3d and 30d, together with 30d^Spd^ flies (GCamp3.0 response averaged across three odor exposures from 6–7 animals). (f) Maximum change in GCamp3.0 fluorescence (ΔF*/F* in %) in response to 3-Oct in PN boutons of 3d and 30d as well as 30d^Spd^ flies (GCamp3.0 response averaged across three odor exposures from 6–7 animals; Kruskal-Wallis test). (g) Time course of Ca^2+^ activity induced by MCH (averaged across three odor exposure) in the presynaptic terminals of PNs within calyx neuropil of 3d and 30d flies, together with 30d^Spd^ flies (GCamp3.0 response averaged across three odor exposures from 6–7 animals). (h) Maximum change in GCamp3.0 fluorescence (ΔF*/F* in %) in response to MCH in PN boutons of 3d, 30d, and 30d^Spd^ flies (GCamp3.0 response averaged across three odor exposures from 6–7 animals; Kruskal-Wallis test). The grey bars indicate the duration of the odor stimuli. ns = not significant, *p* ≥ 0.05. Underlying data is shown in S1 Data.
(TIF)

S4 Fig Decay time constant (τ) of odor-evoked SynpH response. (a) Overall decay time constant (τ) of SynpH signal in response to 3-Oct (3-Octonal). (b) Fast component of decay time constant (τ) of SynpH signal in response to 3-Oct. (c) Slow component of decay time constant (τ) of SynpH signal in response to 3-Oct. (d) Overall decay time constant (τ) of SynpH signal in response to MCH. (e) Fast component of decay time constant (τ) of SynpH signal in response to MCH. (f) Slow component of decay time constant (τ) of SynpH signal in response to MCH. (*n* = 6–7 flies; Kruskal-Wallis test). ns = not significant, *p* ≥ 0.05. Underlying data is shown in S1 Data.
(TIF)

S5 Fig KCl-induced changes in fluorescence of SynpH within the calyx neuropil. (a) SynpH expressed in the PNs and imaged within the calyx region. The two rings indicate the region of interest (calyx neuropil) and background region used for analysis. Scale bar: 50 μm. (b) KCl-induced release of SVs, measured by changes in fluorescence (ΔF*/F* in %) of SynpH of a single fly over time. (c) Maximum change in fluorescence (ΔF*/F* in %) of SynpH response to KCl in 3d, 30d, and 30d^Spd^ flies (*n* = 5–6 flies; Kruskal-Wallis test). ns = not significant, *p* ≥ 0.05. Underlying data is shown in S1 Data.
(TIF)

S6 Fig Imaging of SynpH at KC-to-MBON synapses to measure odor-evoked SV release. (a) Time course of SynpH activity induced by 3-Oct in the presynaptic terminals of KCs within the horizontal lobe of mushroom body of 3d, 30d, and 30d^Spd^ animals (SynpH response averaged across three odor exposures from 6–7 flies). (b) Maximum change in SynpH fluorescence (ΔF*/F* in %) in response to 3-Oct within the presynaptic terminals of KCs of 3d, 30d, and 30d^Spd^ flies (SynpH response averaged across three odor exposures from 6–7 flies; Kruskal-Wallis test with Dunn’s multiple comparison test, *p*-values were subject to Bonferroni correction). (c) Time course of SynpH activity induced by MCH in the presynaptic terminals KCs within the horizontal lobe of mushroom body of 3d, 30d, and 30d^Spd^ animals (SynpH response averaged across three odor exposures from 6–7 flies) (d) Maximum change in SynpH fluorescence (ΔF*/F* in %) in response to MCH within the presynaptic terminals of KCs of 3d, 30d, and 30d^Spd^ flies (SynpH response averaged across three odor exposures from 6–7 flies; Kruskal-Wallis test with Dunn’s multiple comparison test, *p*-values were subject to Bonferroni correction). * *p* < 0.05, ** *p* < 0.01, ns = not significant, *p* ≥ 0.05. Underlying data is shown in S1 Data.
(TIF)

S7 Fig Analysis of Syb in wild-type brains. (a–c) Adult brains of 3d, 30d and 30d^Spd^ *w*^*1118*^ flies immunostained for Syb. Scale bar: 50 μm. (d) Quantification of Syb intensity within the central brain region normalized to 3d flies (*n* = 6–9 independent brains; Kruskal-Wallis test). ns = not significant, *p* ≥ 0.05. Underlying data is shown in S1 Data.
(TIF)

S8 Fig BRP and RBP increase progressively with age. (a–d) Adult brains of 3d, 10d, 20d, and 30d *w*^*1118*^ flies immunostained for BRP (using Nc82 and N-terminal antibodies) and RBP. Scale bar: 50 μm (e–g) Quantification of BRP (using Nc82 and N-terminal antibodies) and RBP intensities within the central brain region normalized to 3d flies (*n* = 10–12 independent brains; Kruskal-Wallis test with Dunn’s multiple comparison test, *p*-values were subject to Bonferroni correction). ** *p* < 0.01, *** *p* < 0.001, ns = not significant, *p* ≥ 0.05. Underlying data is shown in S1 Data.
(TIF)

S9 Fig Analysis of the endogenous expression of BRP^GFP^ in adult brains. (a–c) Adult brains of 3d- and 30d-BRP(83-ex13)^GFP^ flies, and 30d^Spd-^ BRP(83-ex13)^GFP^ flies (BRP^GFP^). Brains were fixed in 5% PFA and scanned for GFP signal. Scale bar: 50 μm. (d) Quantification of GFP signal within the central brain region normalized to 3d flies (*n* = 9–18 independent brains; Kruskal-Wallis test with Dunn’s multiple comparison test, *p*-values were subject to Bonferroni correction). * *p* < 0.05, ** *p* < 0.01, *** *p* < 0.001. Underlying data is shown in S1 Data.
(TIF)

S10 Fig Analysis of Ca^2+^ channel and BRP in wild-type brains. (a–c) Mushroom body calyx of 3d, 30d, and 30d^Spd^ flies expressing GFP-labeled genomic construct of α1 subunit Cacophony (Cac^GFP^) and immunostained for GFP as well as BRP (corresponding single z-planes are shown). Scale bar: 10 μm. (d,e) Quantification of signal intensity of Cac^GFP^ (using anti-GFP) and BRP (using Nc82) in the calyx region normalized to 3d flies (*n* = 7–9 independent calyces; Kruskal-Wallis test with Dunn’s multiple comparison test, *p*-values were subject to Bonferroni correction). ** *p* < 0.01, ns = not significant, *p* ≥ 0.05. Underlying data is shown in S1 Data.
(TIF)

S11 Fig Analysis of for Unc13 and BRP in wild-type brains. (a–c) Adult brains of 3d and 30d *w*^*1118*^ flies, together with 30d^Spd^ *w*^*1118*^ flies, immunostained for BRP (using Nc82 and N-terminal antibody) and Unc13. Scale bar: 50 μm. (d–f) Quantification of signal intensity of the proteins in the central brain region normalized to 3d flies (*n* = 10–15 independent brains; Kruskal-Wallis test with Dunn’s multiple comparison test, *p*-values were subject to Bonferroni correction). * *p* < 0.05, ** *p* < 0.01, *** *p* < 0.001. Underlying data is shown in S1 Data.
(TIF)

S12 Fig EM of PN-to-KC synapses. Electron micrographs revealed that the alignment of the plasma membrane, with evident increase in extracellular spacing between cellular elements, to be affected in 30d *w*^*1118*^ flies, when compared to 3d or 30d^Spd^ *w*^*1118*^ flies. Scale bar: 500 nm. The arrowheads point to the alignment of the plasma membrane between subcellular entities.
(TIF)

S13 Fig STED analysis of BRP ring diameter at PN-to-KC synapses. Examples of confocal and STED images of BRP spots within the calyx region of 3d and 30d *w*^*1118*^ flies, together with 30d^Spd^ *w*^*1118*^ flies. Scale bar: 500 nm. These calyces were also stained for Drep2, a protein found highly enriched in dendritic claws of KCs, allowing the quantification of the diameter of BRP spots that mark the synapse between PNs and KCs.
(TIF)

S14 Fig Effect of removing one-copy of BRP on memory formation. (a–d) Adult brains of 3d and 30d *brp69/+* (1xBRP) flies together with age-matched controls (2xBRP), immunostained for BRP (using Nc82 and N-terminal antibody), and RBP. Scale bar: 50 μm. (e, f) Quantification of BRP (using N-terminal antibody) and RBP intensity within the central brain region normalized to 3d flies (9–10 independent brains; Kruskal-Wallis test with Dunn’s multiple comparison test, *p*-values were subject to Bonferroni correction). (g) Aversive associative memory performance 3 min after training (STM) of *brp69/+* (1xBRP) flies compared to wild-type (2xBRP) flies (*n* = 7–12; Kruskal-Wallis test with Dunn’s multiple comparison test, *p*-values were subject to Bonferroni correction). * *p* < 0.05, ** *p* < 0.01, ns = not significant, *p* ≥ 0.05. Underlying data is shown in S1 Data.
(TIF)

S15 Fig Acetylation and mass spectroscopy. (a) BRP sequence with acetylated peptide fragments (yellow) and lysine sites positive for acetylation (red) identified through mass spectroscopy. (b) Position of possible lysine residues that undergo (de)acetylation within BRP.
(TIF)

S16 Fig KCl-induced changes in fluorescence of Homer GCamp3.0 within the calyx neuropil. (a) Homer GCamp3.0 expressed in the dendritic claws of KCs and imaged within the calyx region. The two rings indicate the region of interest (calyx neuropil) and background region used for analysis. Scale bar: 50 μm. (b) KCl-induced influx of postsynaptic Ca2+ ion, measured by changes in fluorescence (ΔF/F in %) of Homer GCamp3.0 of a single fly over time. (c) Maximum change in fluorescence (ΔF/F in %) of Homer GCamp3.0 response to KCl in 3d, 30d, and 30d^Spd^ flies (*n* = 8–9 flies; Kruskal-Wallis test). ns = not significant, *p* ≥ 0.05. Underlying data is shown in S1 Data.
(TIF)

S17 Fig Empirical cumulative distribution functions for postsynaptic Homer-GCaMP3.0 response during odor stimulation. Empirical cumulative distribution functions for 3-Oct and MCH, as used in the Kolmogorov-Smirnov test. Only the GCaMP3 response during odor presentation (seconds 1–3, grey bars in Fig 3K and 3M) was used. Two-sided Kolmogorov-Smirnov tests were conducted for the analysis of difference. The differences for 3-Oct were not significant. The differences for MCH between 3d and 30d (**), as well as between 30d and 30d^Spd^ (*) were significant after Bonferroni correction for three groups. * *p* < 0.05, ** *p* < 0.01. Underlying data is shown in S1 Data.
(TIF)

S18 Fig Calyx neuropil from wild-type brains immunostained for Drep2 and BRP. (a–c) Mushroom body calyx from adult brains of 3d and 30d *w*^*1118*^ flies, together with 30d^Spd^ *w*^*1118*^ flies immunostained for Drep2 and BRP (using Nc82 as well as N-terminal antibodies) (corresponding single z-planes are shown). Scale bar: 10 μm. (d–f) Quantification of signal intensity of these proteins in the calyx region normalized to 3d flies (*n* = 10 independent brains; Kruskal-Wallis test with Dunn’s multiple comparison test, *p*-values were subject to Bonferroni correction). ** *p* < 0.01, *** *p* < 0.001, ns = not significant, *p* ≥ 0.05. Underlying data is shown in S1 Data.
(TIF)

S19 Fig Autophagy required for spermidine-mediated suppression of age-associated increase in BRP levels. (a–d) Comparison of BRP signal intensity (using Nc82 and N-terminal antibodies) in brains of 3d *w*^*1118*^ control animals, 3d and 20-d old (20d) *atg7*^*-/-*^ flies, raised either on normal or spermidine-supplemented food. Scale bar: 50 μm. (e–f) Quantification of BRP within the central brain region of young (3d) and old (20d) *atg7*^*-/-*^ mutants normalized to 3d *w*^*1118*^ flies (*n* = 9–12 independent brains; Kruskal-Wallis test with Dunn’s multiple comparison test, *p*-values were subject to Bonferroni correction). ** *p* < 0.01, ns = not significant, *p* ≥ 0.05. Underlying data is shown in S1 Data.
(TIF)

S1 Table Aversive odor avoidance and shock reactivity in different genotypes. Underlying data is shown in S1 Data.
(TIF)

## Abbreviations

AMI: age-dependent memory impairment
ARM: anesthesia-resistant memory
ASM: anesthesia-sensitive memory
AZ: active zone
BRP: Bruchpilot
EM: electron microscopy
GFP: green fluorescent protein
HDAC6: histone deacetylase-6
ITM: intermediate-term memory
KC: kenyon cell
MBON: mushroom body output neuron
MCH: 4-methylcyclehexanol
PN: projection neuron
RBP: rim-binding protein
STED: stimulated emission depletion microscopy
STM: short-term memory
SV: synaptic vesicle
Syb: Synaptobrevin
SynpH: SynaptopHluorin
2xBRP: two-copy BRP
3d: 3-day old flies
3-Oct: 3-Octanol
4xBRP: four-copy BRP
30d: 30-day-old flies
30d^Spd^: 30-day-old flies treated with spermidine
