# Supplementary figures and images for: Spermidine Suppresses Age-Associated Memory Impairment by Preventing Adverse Increase of Presynaptic Active Zone Size and Release
Source: PLoS Biol. 2016 Sep 29;14(9):e1002563. doi: 10.1371/journal.pbio.1002563 (PMC5042543; doi:10.1371/journal.pbio.1002563)

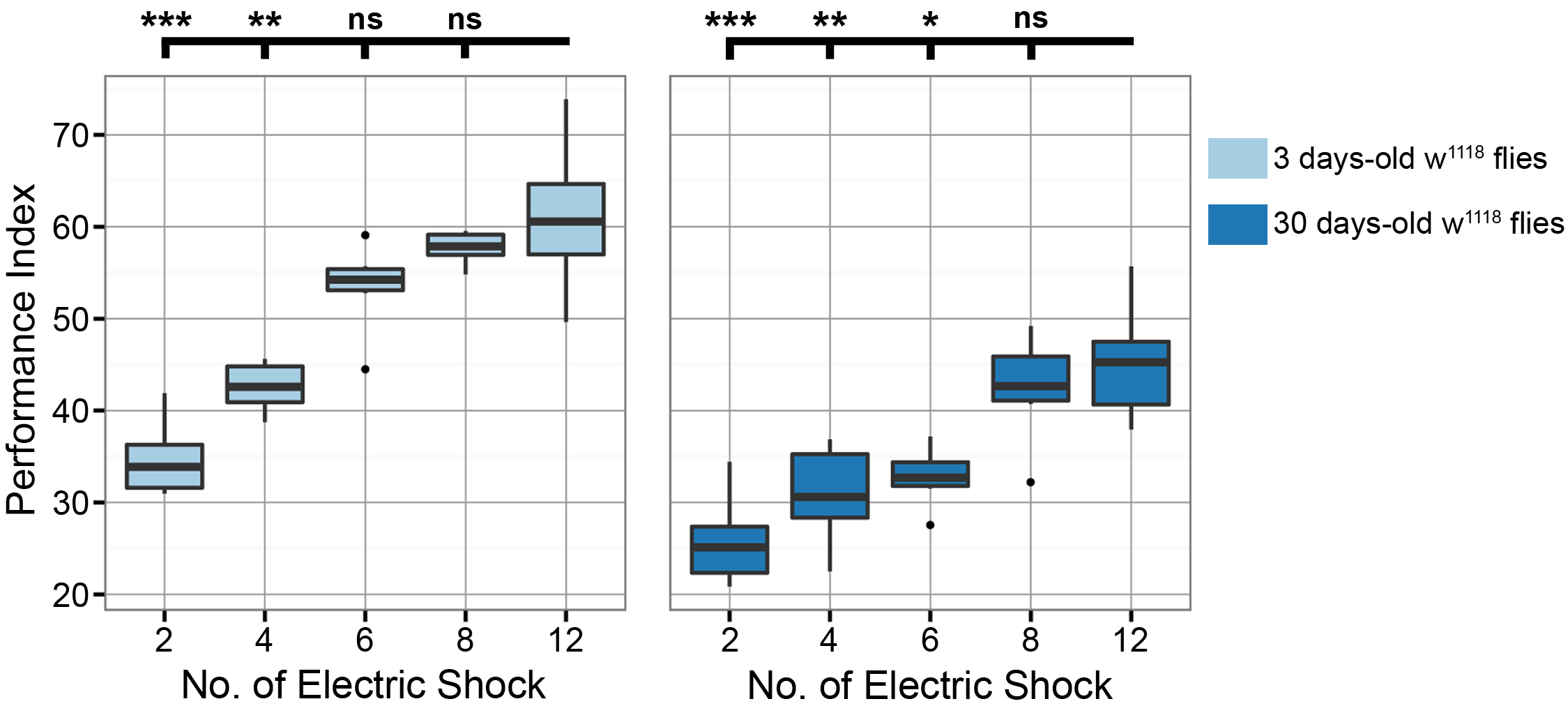

Supplement: S1 Fig — STM index plotted against shock number as experienced during training sessions with 120 V DC in 3d (light blue bars) and 30d (dark blue bars) wild-type w1118 flies (n = 6–8; Kruskal-Wallis test with Dunn’s multiple comparison test, p-values were subject to Bonferroni correction). * p < 0.05, ** p < 0.01, *** p < 0.001, ns = not significant, p ≥ 0.05. Underlying data is shown in S1 Data. (TIF) [file pbio.1002563.s002.tif]

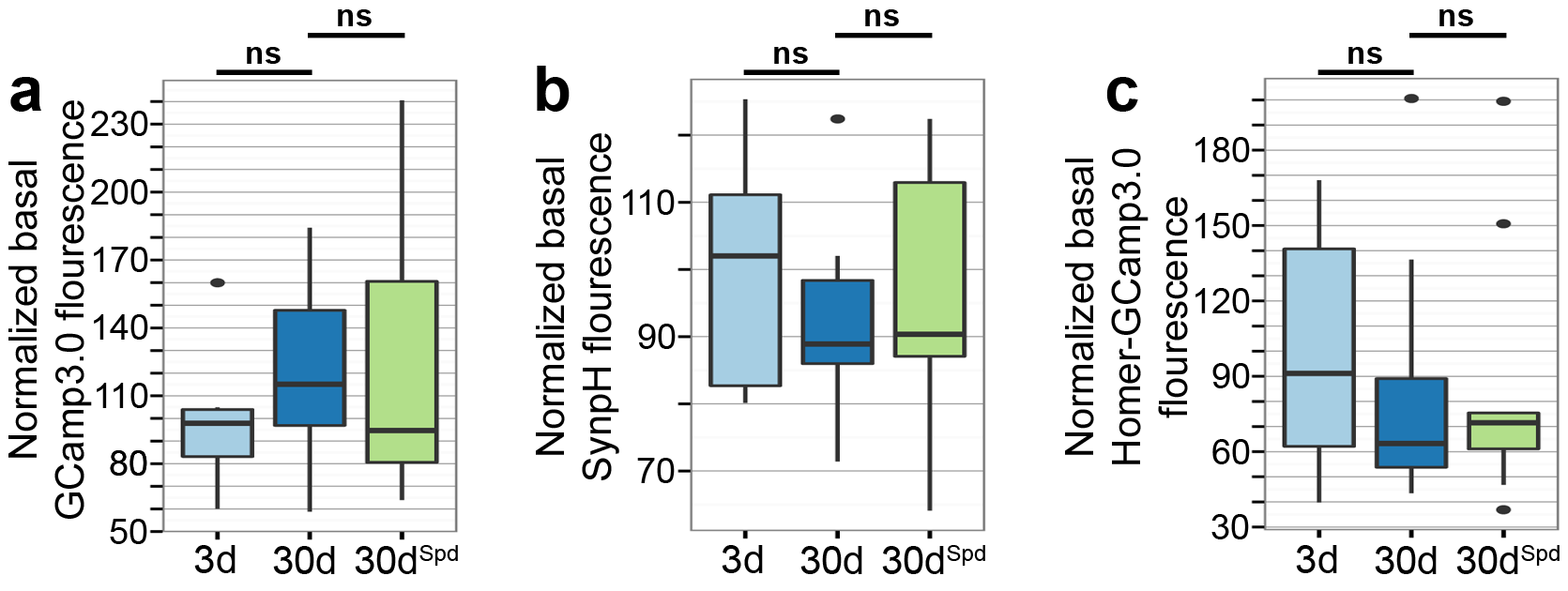

Supplement: S2 Fig — (a) Quantification of levels of GCamp3.0 in the PN terminals within the calyx region normalized to 3d flies (n = 6–7 independent calyces; Kruskal-Wallis test). (b) Quantification of levels of SynaptopHlourin (SynpH) in the PN terminals within the calyx region normalized to 3d flies (n = 7–12 independent calyces; Kruskal-Wallis test). (c) Quantification of levels of Homer-GCamp3.0 in the dendritic claws of KCs within the calyx region normalized to 3d flies (n = 10–12 independent calyces; Kruskal-Wallis test). ns = not significant, p ≥ 0.05. Underlying data is shown in S1 Data. (TIF) [file pbio.1002563.s003.tif]

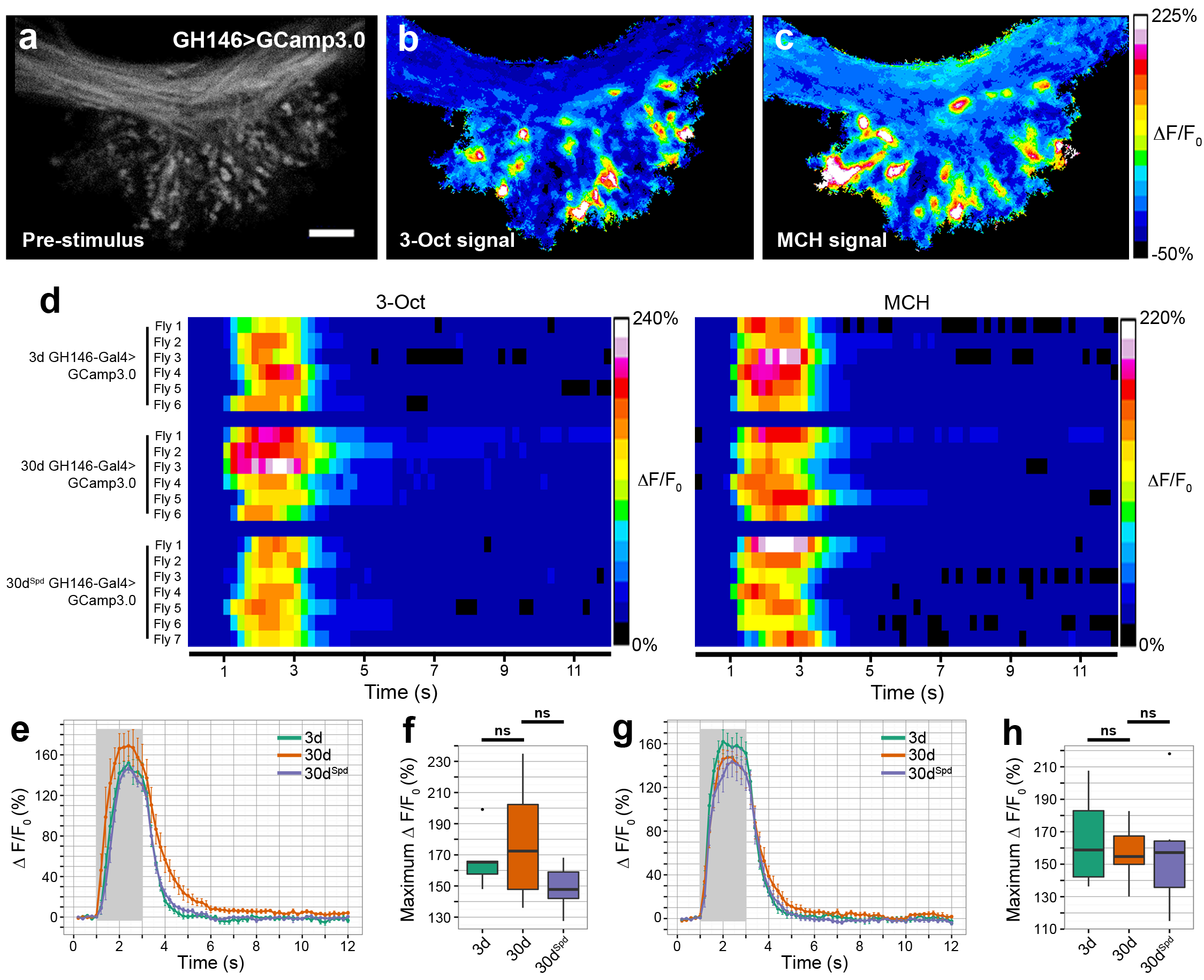

Supplement: S3 Fig — (a) Expression of GCaMP3.0 in the PNs and imaged within the calyx neuropil. (b, c) False color-coded image of Ca2+ activity within the presynaptic terminals of PNs in response to 3-Oct and MCH shown in (a). Warm colors indicate high levels, while cold colors low levels or no Ca2+ activity. The numbers indicate changes in fluorescence (ΔF/F in %). Scale bar: 10 μm. (d) Odor-evoked Ca2+ activity, measured by changes in fluorescence of Gamp3.0, of an individual fly over time, shown as false colors in the presynaptic terminal of PNs in calyx region, in response to the odorants 3-Oct and MCH. (GCamp3.0 response averaged across three odor exposures from 6–7 animals). (e) Time course of Ca2+ activity induced by 3-Oct (averaged across three odor exposure) in the presynaptic terminals of PNs within calyx neuropil of 3d and 30d, together with 30dSpd flies (GCamp3.0 response averaged across three odor exposures from 6–7 animals). (f) Maximum change in GCamp3.0 fluorescence (ΔF/F in %) in response to 3-Oct in PN boutons of 3d and 30d as well as 30dSpd flies (GCamp3.0 response averaged across three odor exposures from 6–7 animals; Kruskal-Wallis test). (g) Time course of Ca2+ activity induced by MCH (averaged across three odor exposure) in the presynaptic terminals of PNs within calyx neuropil of 3d and 30d flies, together with 30dSpd flies (GCamp3.0 response averaged across three odor exposures from 6–7 animals). (h) Maximum change in GCamp3.0 fluorescence (ΔF/F in %) in response to MCH in PN boutons of 3d, 30d, and 30dSpd flies (GCamp3.0 response averaged across three odor exposures from 6–7 animals; Kruskal-Wallis test). The grey bars indicate the duration of the odor stimuli. ns = not significant, p ≥ 0.05. Underlying data is shown in S1 Data. (TIF) [file pbio.1002563.s004.tif]

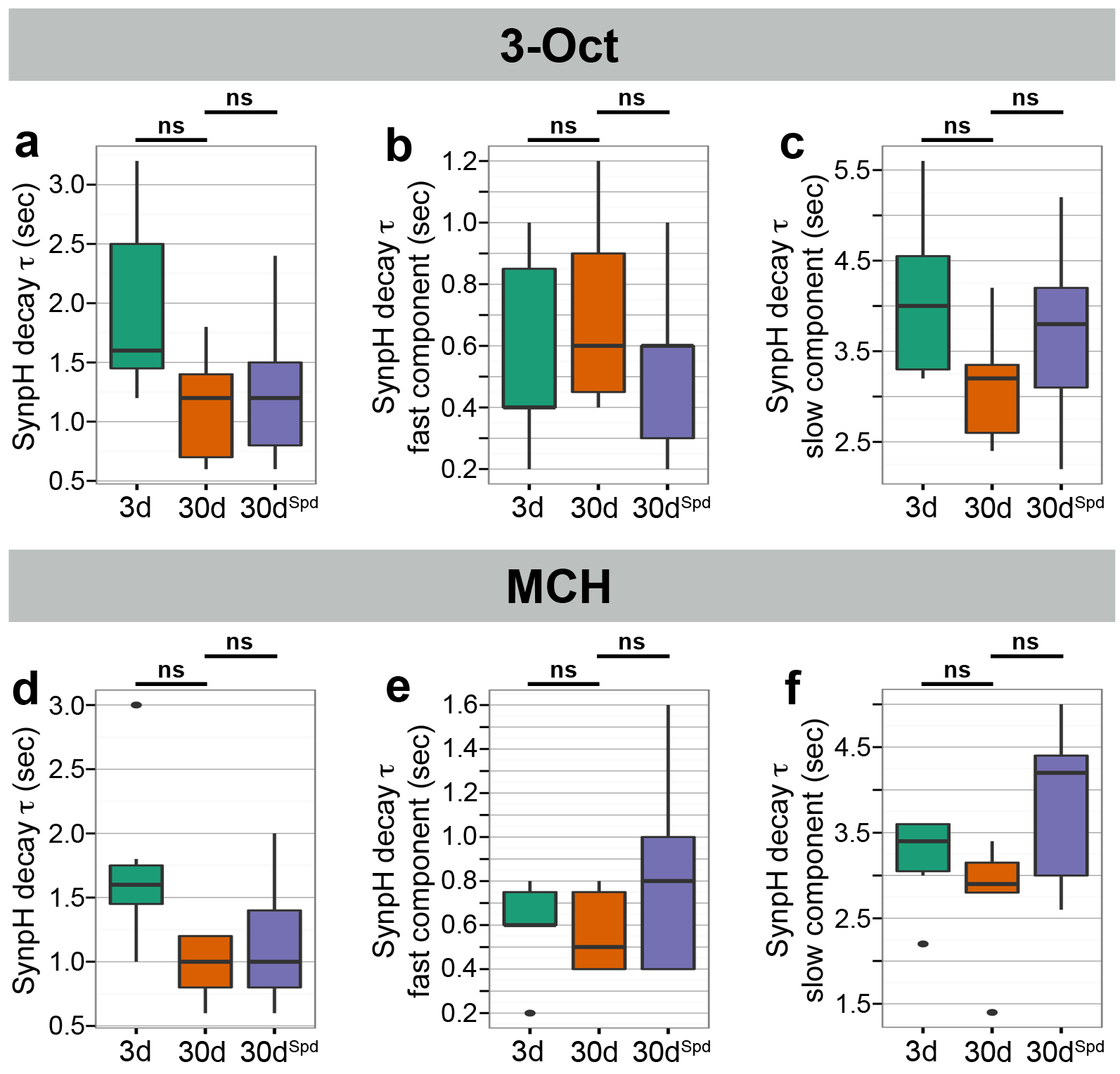

Supplement: S4 Fig — (a) Overall decay time constant (τ) of SynpH signal in response to 3-Oct (3-Octonal). (b) Fast component of decay time constant (τ) of SynpH signal in response to 3-Oct. (c) Slow component of decay time constant (τ) of SynpH signal in response to 3-Oct. (d) Overall decay time constant (τ) of SynpH signal in response to MCH. (e) Fast component of decay time constant (τ) of SynpH signal in response to MCH. (f) Slow component of decay time constant (τ) of SynpH signal in response to MCH. (n = 6–7 flies; Kruskal-Wallis test). ns = not significant, p ≥ 0.05. Underlying data is shown in S1 Data. (TIF) [file pbio.1002563.s005.tif]

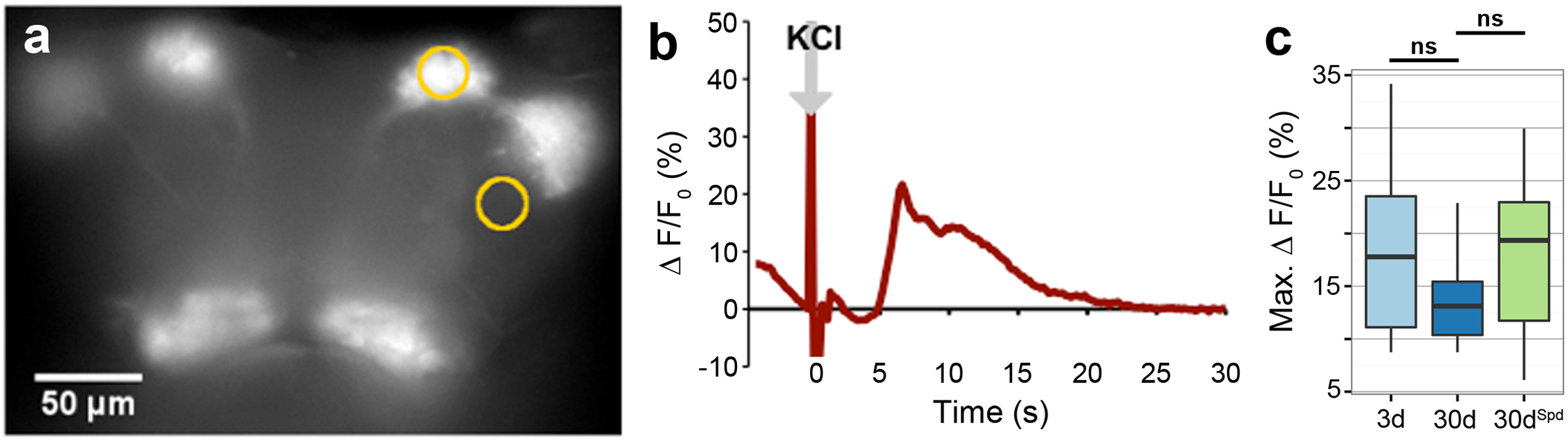

Supplement: S5 Fig — (a) SynpH expressed in the PNs and imaged within the calyx region. The two rings indicate the region of interest (calyx neuropil) and background region used for analysis. Scale bar: 50 μm. (b) KCl-induced release of SVs, measured by changes in fluorescence (ΔF/F in %) of SynpH of a single fly over time. (c) Maximum change in fluorescence (ΔF/F in %) of SynpH response to KCl in 3d, 30d, and 30dSpd flies (n = 5–6 flies; Kruskal-Wallis test). ns = not significant, p ≥ 0.05. Underlying data is shown in S1 Data. (TIF) [file pbio.1002563.s006.tif]

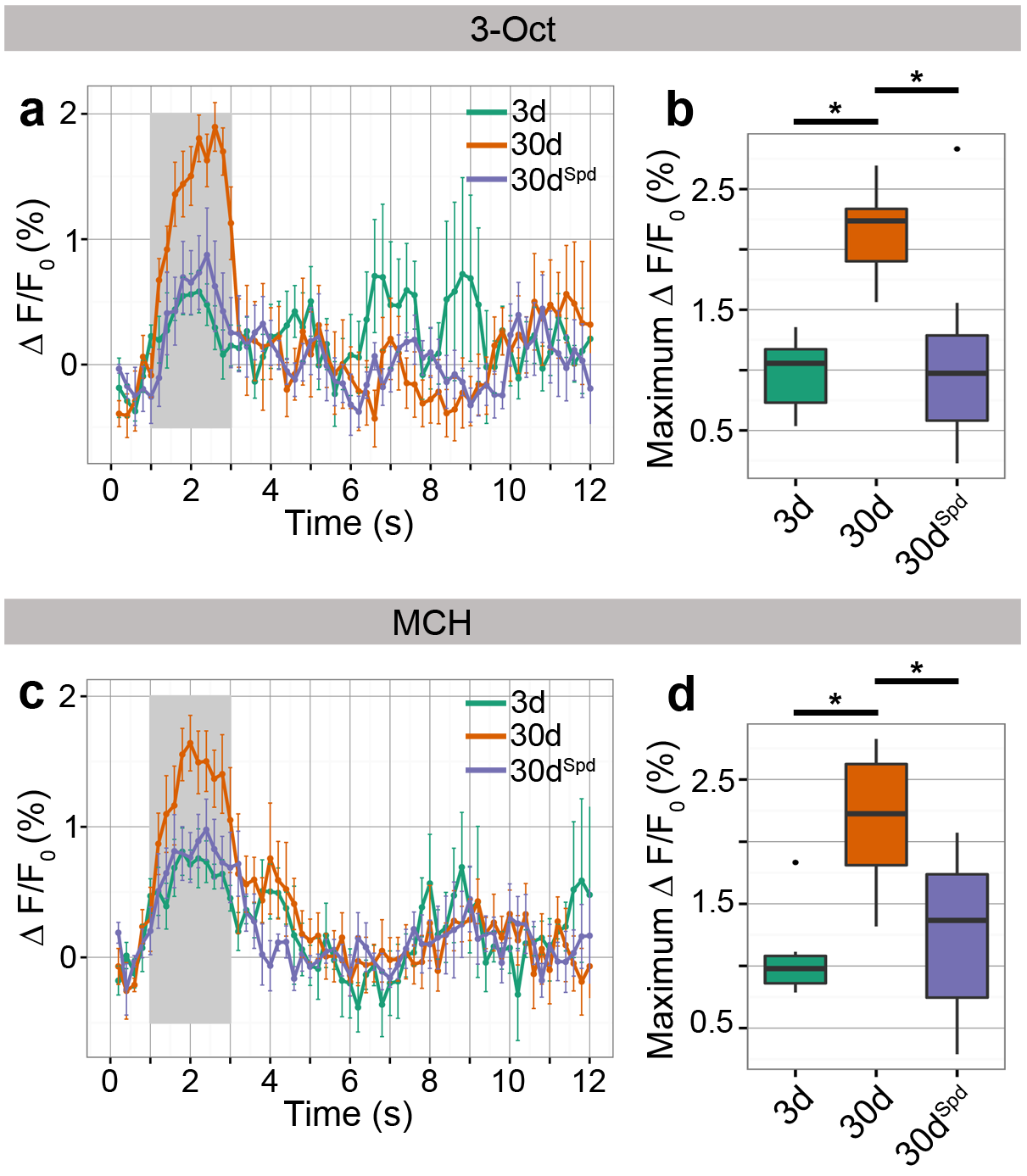

Supplement: S6 Fig — (a) Time course of SynpH activity induced by 3-Oct in the presynaptic terminals of KCs within the horizontal lobe of mushroom body of 3d, 30d, and 30dSpd animals (SynpH response averaged across three odor exposures from 6–7 flies). (b) Maximum change in SynpH fluorescence (ΔF/F in %) in response to 3-Oct within the presynaptic terminals of KCs of 3d, 30d, and 30dSpd flies (SynpH response averaged across three odor exposures from 6–7 flies; Kruskal-Wallis test with Dunn’s multiple comparison test, p-values were subject to Bonferroni correction). (c) Time course of SynpH activity induced by MCH in the presynaptic terminals KCs within the horizontal lobe of mushroom body of 3d, 30d, and 30dSpd animals (SynpH response averaged across three odor exposures from 6–7 flies) (d) Maximum change in SynpH fluorescence (ΔF/F in %) in response to MCH within the presynaptic terminals of KCs of 3d, 30d, and 30dSpd flies (SynpH response averaged across three odor exposures from 6–7 flies; Kruskal-Wallis test with Dunn’s multiple comparison test, p-values were subject to Bonferroni correction). * p < 0.05, ** p < 0.01, ns = not significant, p ≥ 0.05. Underlying data is shown in S1 Data. (TIF) [file pbio.1002563.s007.tif]

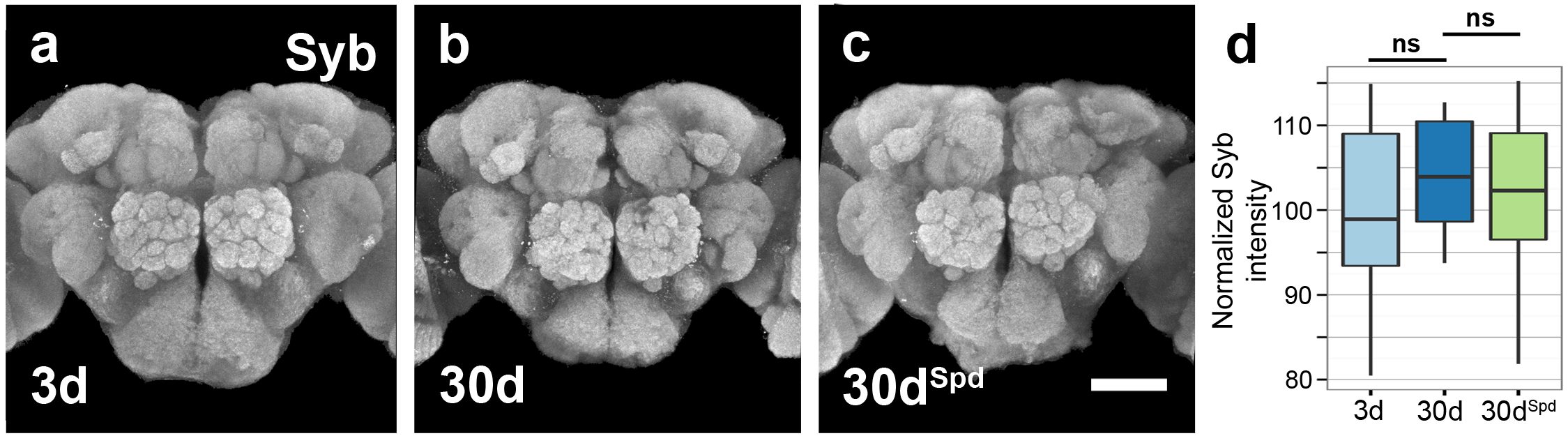

Supplement: S7 Fig — (a–c) Adult brains of 3d, 30d and 30dSpd w1118 flies immunostained for Syb. Scale bar: 50 μm. (d) Quantification of Syb intensity within the central brain region normalized to 3d flies (n = 6–9 independent brains; Kruskal-Wallis test). ns = not significant, p ≥ 0.05. Underlying data is shown in S1 Data. (TIF) [file pbio.1002563.s008.tif]

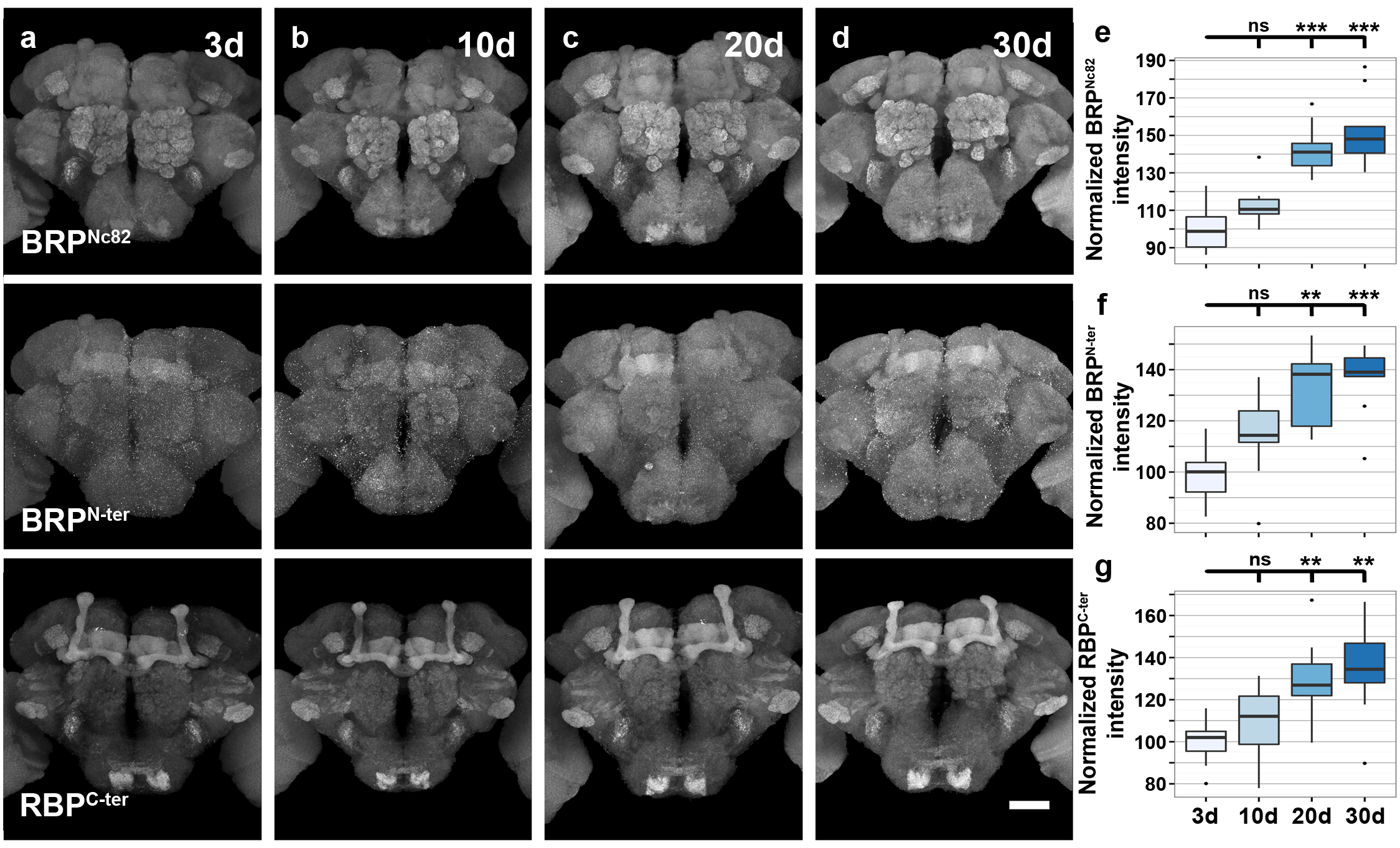

Supplement: S8 Fig — (a–d) Adult brains of 3d, 10d, 20d, and 30d w1118 flies immunostained for BRP (using Nc82 and N-terminal antibodies) and RBP. Scale bar: 50 μm (e–g) Quantification of BRP (using Nc82 and N-terminal antibodies) and RBP intensities within the central brain region normalized to 3d flies (n = 10–12 independent brains; Kruskal-Wallis test with Dunn’s multiple comparison test, p-values were subject to Bonferroni correction). ** p < 0.01, *** p < 0.001, ns = not significant, p ≥ 0.05. Underlying data is shown in S1 Data. (TIF) [file pbio.1002563.s009.tif]

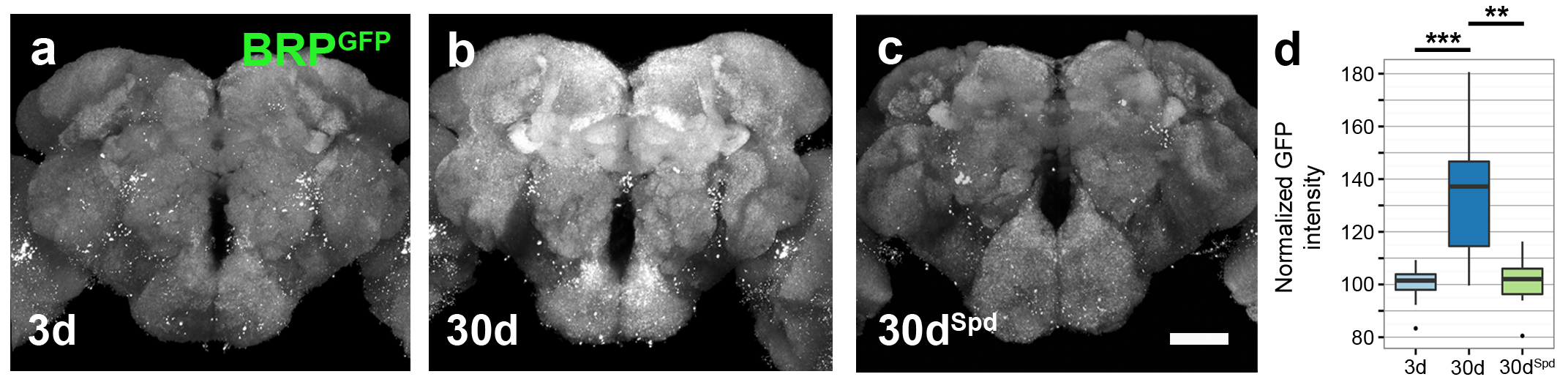

Supplement: S9 Fig — (a–c) Adult brains of 3d- and 30d-BRP(83-ex13)GFP flies, and 30dSpd- BRP(83-ex13)GFP flies (BRPGFP). Brains were fixed in 5% PFA and scanned for GFP signal. Scale bar: 50 μm. (d) Quantification of GFP signal within the central brain region normalized to 3d flies (n = 9–18 independent brains; Kruskal-Wallis test with Dunn’s multiple comparison test, p-values were subject to Bonferroni correction). * p < 0.05, ** p < 0.01, *** p < 0.001. Underlying data is shown in S1 Data. (TIF) [file pbio.1002563.s010.tif]

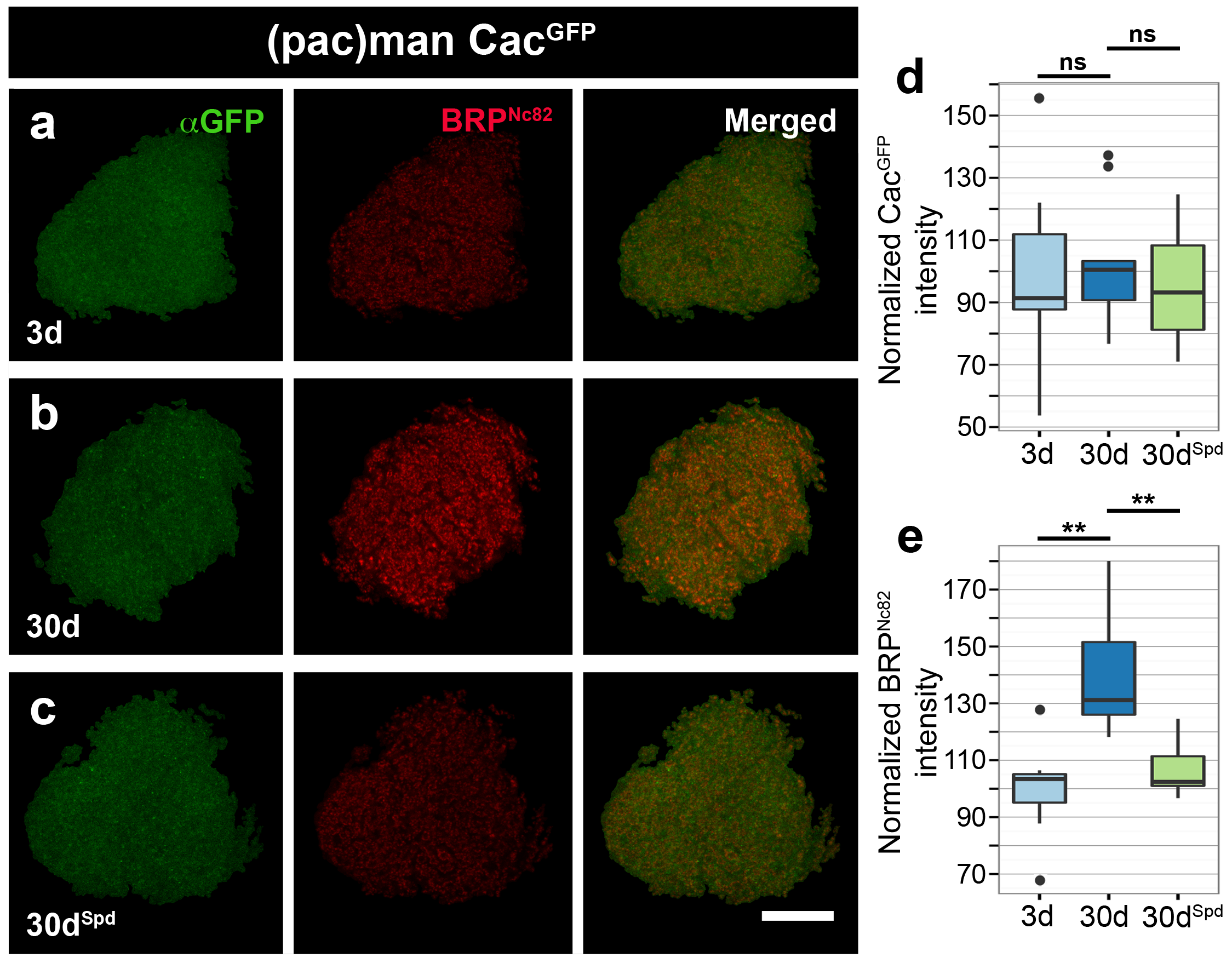

Supplement: S10 Fig — (a–c) Mushroom body calyx of 3d, 30d, and 30dSpd flies expressing GFP-labeled genomic construct of α1 subunit Cacophony (CacGFP) and immunostained for GFP as well as BRP (corresponding single z-planes are shown). Scale bar: 10 μm. (d,e) Quantification of signal intensity of CacGFP (using anti-GFP) and BRP (using Nc82) in the calyx region normalized to 3d flies (n = 7–9 independent calyces; Kruskal-Wallis test with Dunn’s multiple comparison test, p-values were subject to Bonferroni correction). ** p < 0.01, ns = not significant, p ≥ 0.05. Underlying data is shown in S1 Data. (TIF) [file pbio.1002563.s011.tif]

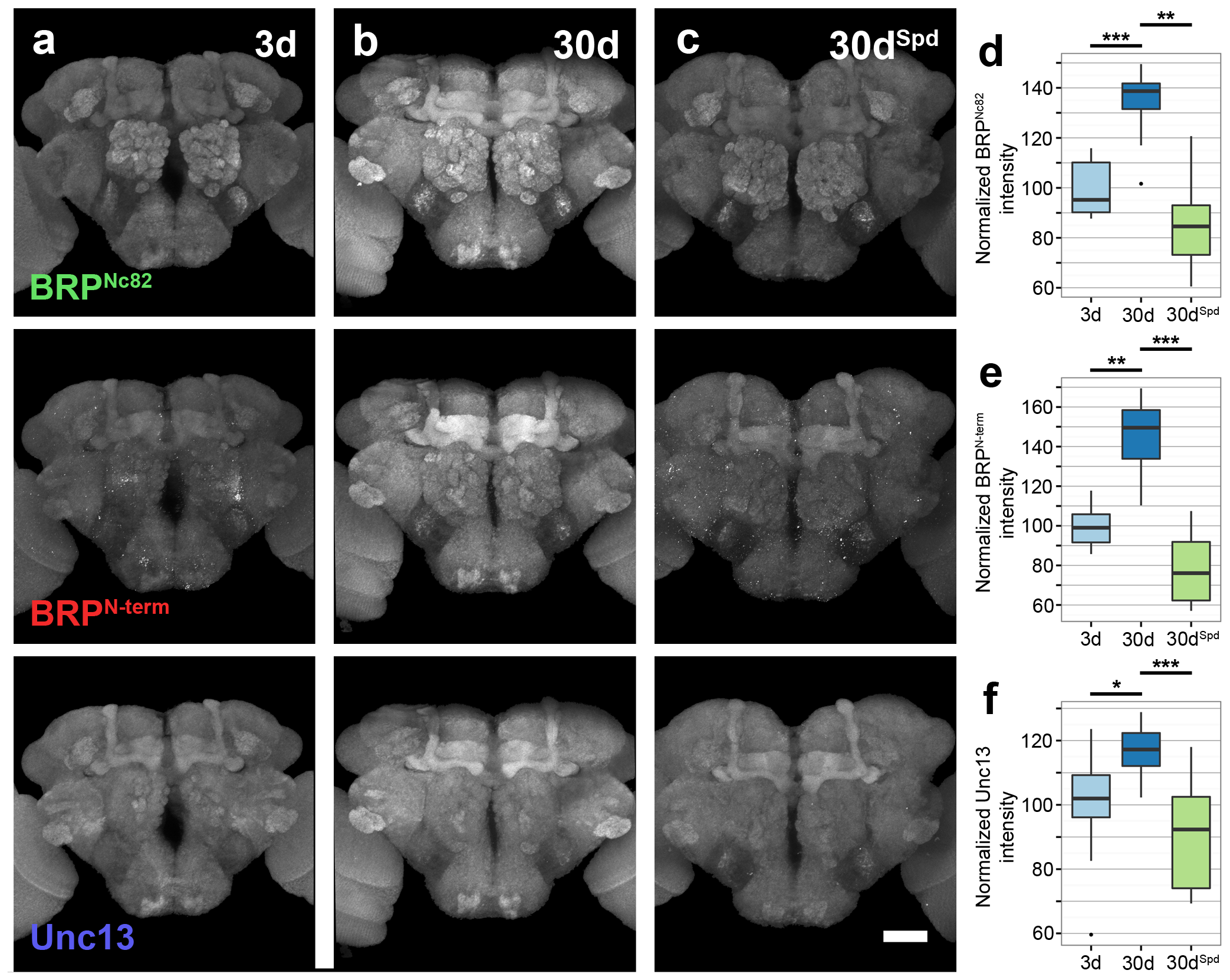

Supplement: S11 Fig — (a–c) Adult brains of 3d and 30d w1118 flies, together with 30dSpd w1118 flies, immunostained for BRP (using Nc82 and N-terminal antibody) and Unc13. Scale bar: 50 μm. (d–f) Quantification of signal intensity of the proteins in the central brain region normalized to 3d flies (n = 10–15 independent brains; Kruskal-Wallis test with Dunn’s multiple comparison test, p-values were subject to Bonferroni correction). * p < 0.05, ** p < 0.01, *** p < 0.001. Underlying data is shown in S1 Data. (TIF) [file pbio.1002563.s012.tif]

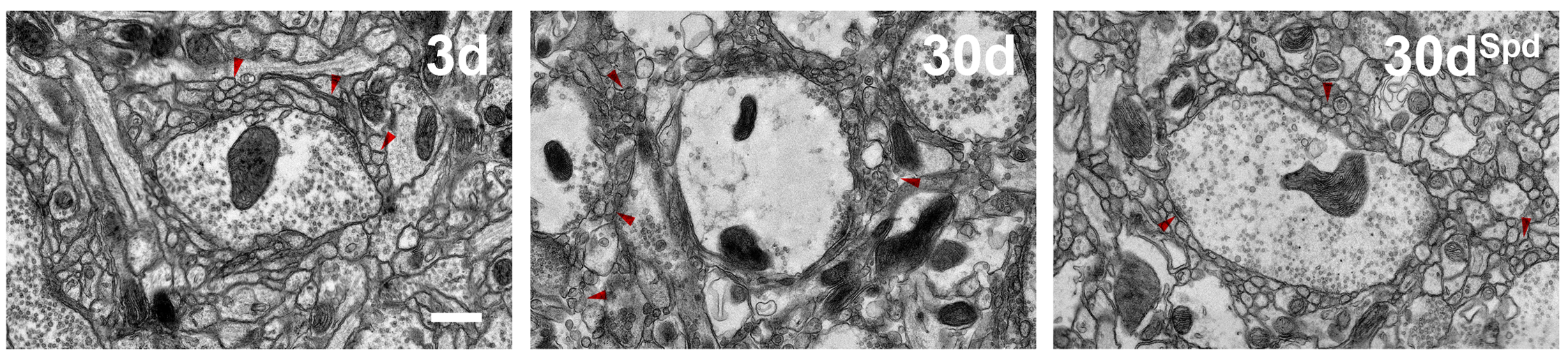

Supplement: S12 Fig — Electron micrographs revealed that the alignment of the plasma membrane, with evident increase in extracellular spacing between cellular elements, to be affected in 30d w1118 flies, when compared to 3d or 30dSpd w1118 flies. Scale bar: 500 nm. The arrowheads point to the alignment of the plasma membrane between subcellular entities. (TIF) [file pbio.1002563.s013.tif]

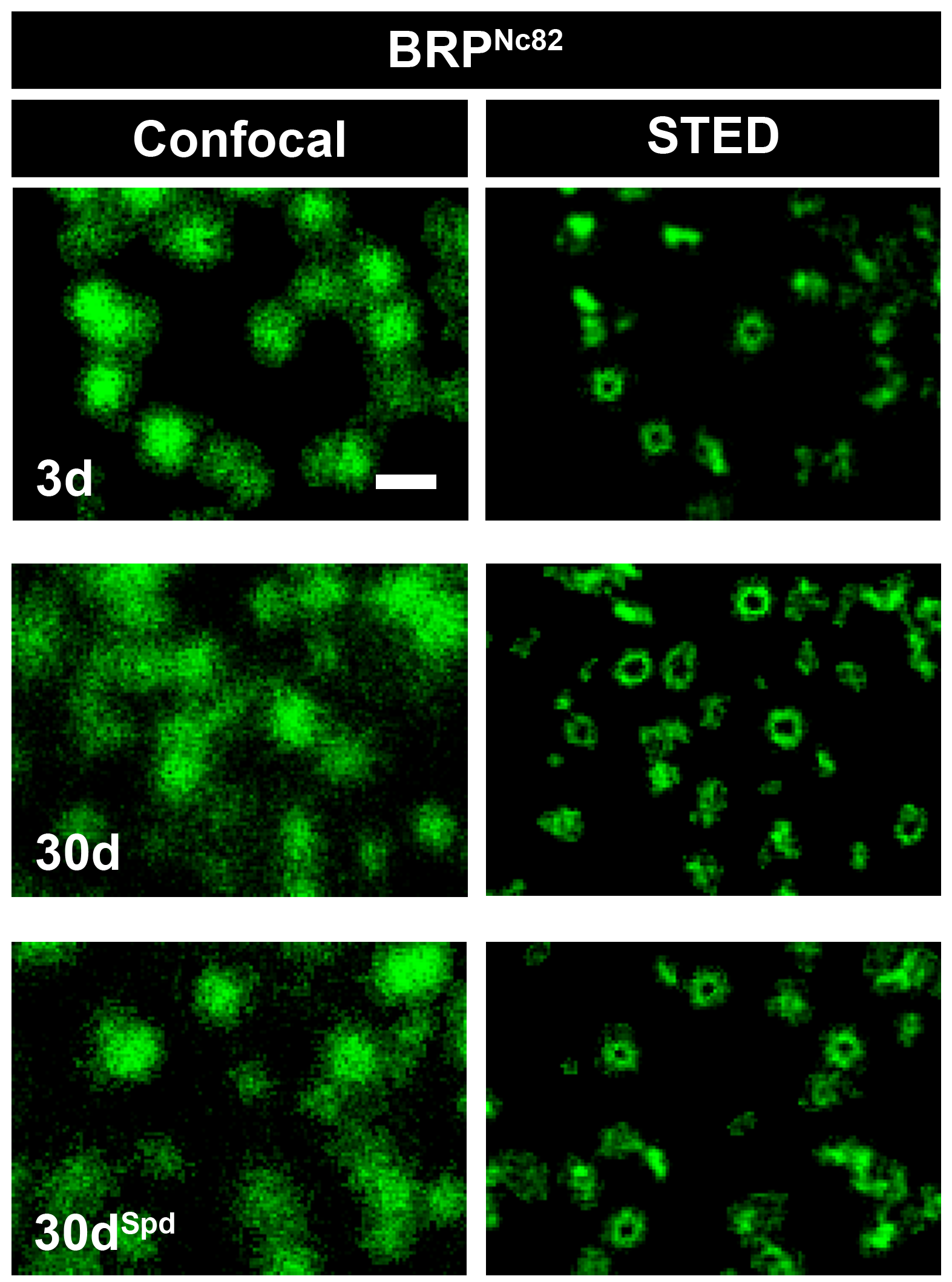

Supplement: S13 Fig — Examples of confocal and STED images of BRP spots within the calyx region of 3d and 30d w1118 flies, together with 30dSpd w1118 flies. Scale bar: 500 nm. These calyces were also stained for Drep2, a protein found highly enriched in dendritic claws of KCs, allowing the quantification of the diameter of BRP spots that mark the synapse between PNs and KCs. (TIF) [file pbio.1002563.s014.tif]

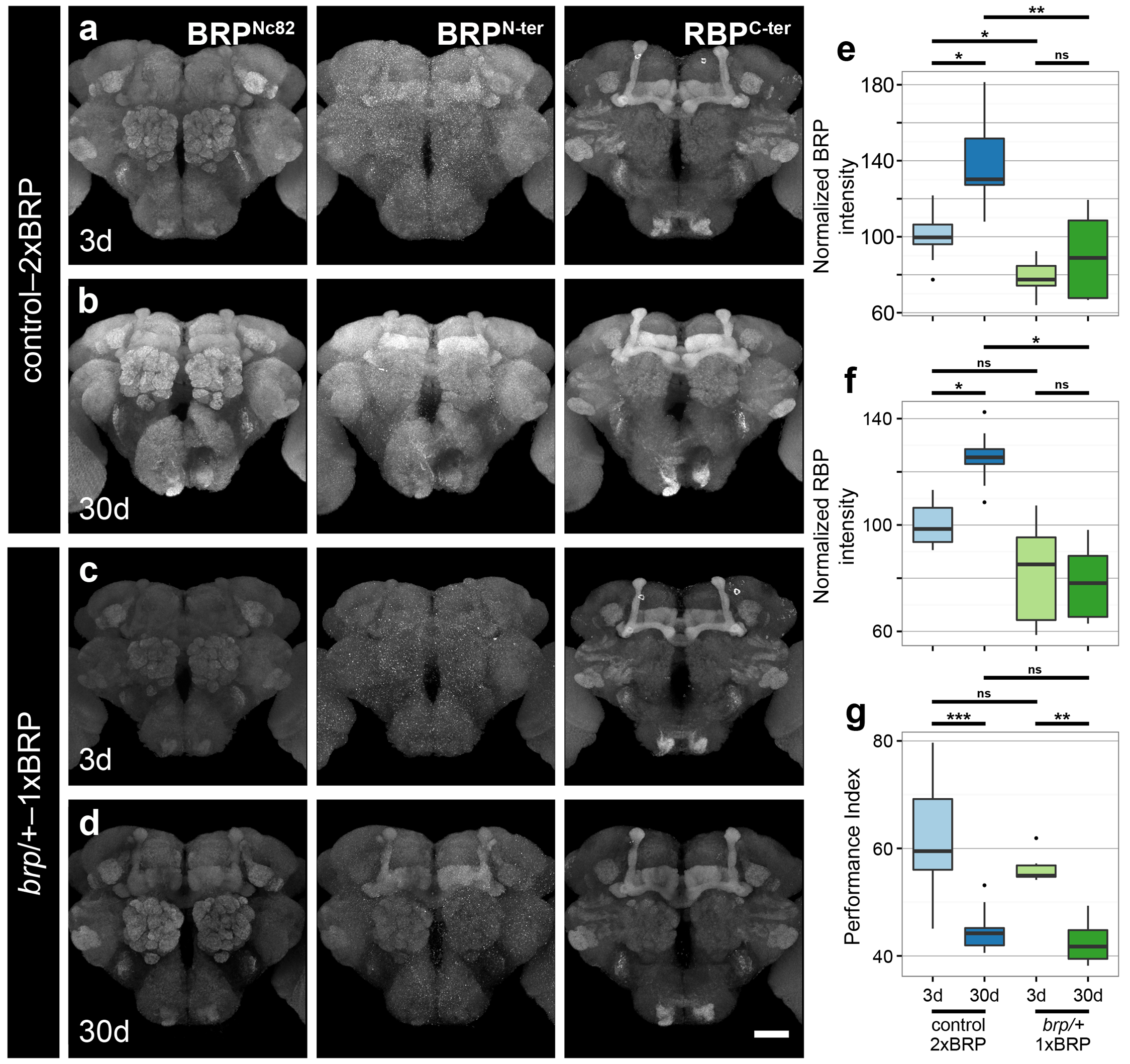

Supplement: S14 Fig — (a–d) Adult brains of 3d and 30d brp69/+ (1xBRP) flies together with age-matched controls (2xBRP), immunostained for BRP (using Nc82 and N-terminal antibody), and RBP. Scale bar: 50 μm. (e, f) Quantification of BRP (using N-terminal antibody) and RBP intensity within the central brain region normalized to 3d flies (9–10 independent brains; Kruskal-Wallis test with Dunn’s multiple comparison test, p-values were subject to Bonferroni correction). (g) Aversive associative memory performance 3 min after training (STM) of brp69/+ (1xBRP) flies compared to wild-type (2xBRP) flies (n = 7–12; Kruskal-Wallis test with Dunn’s multiple comparison test, p-values were subject to Bonferroni correction). * p < 0.05, ** p < 0.01, ns = not significant, p ≥ 0.05. Underlying data is shown in S1 Data. (TIF) [file pbio.1002563.s015.tif]

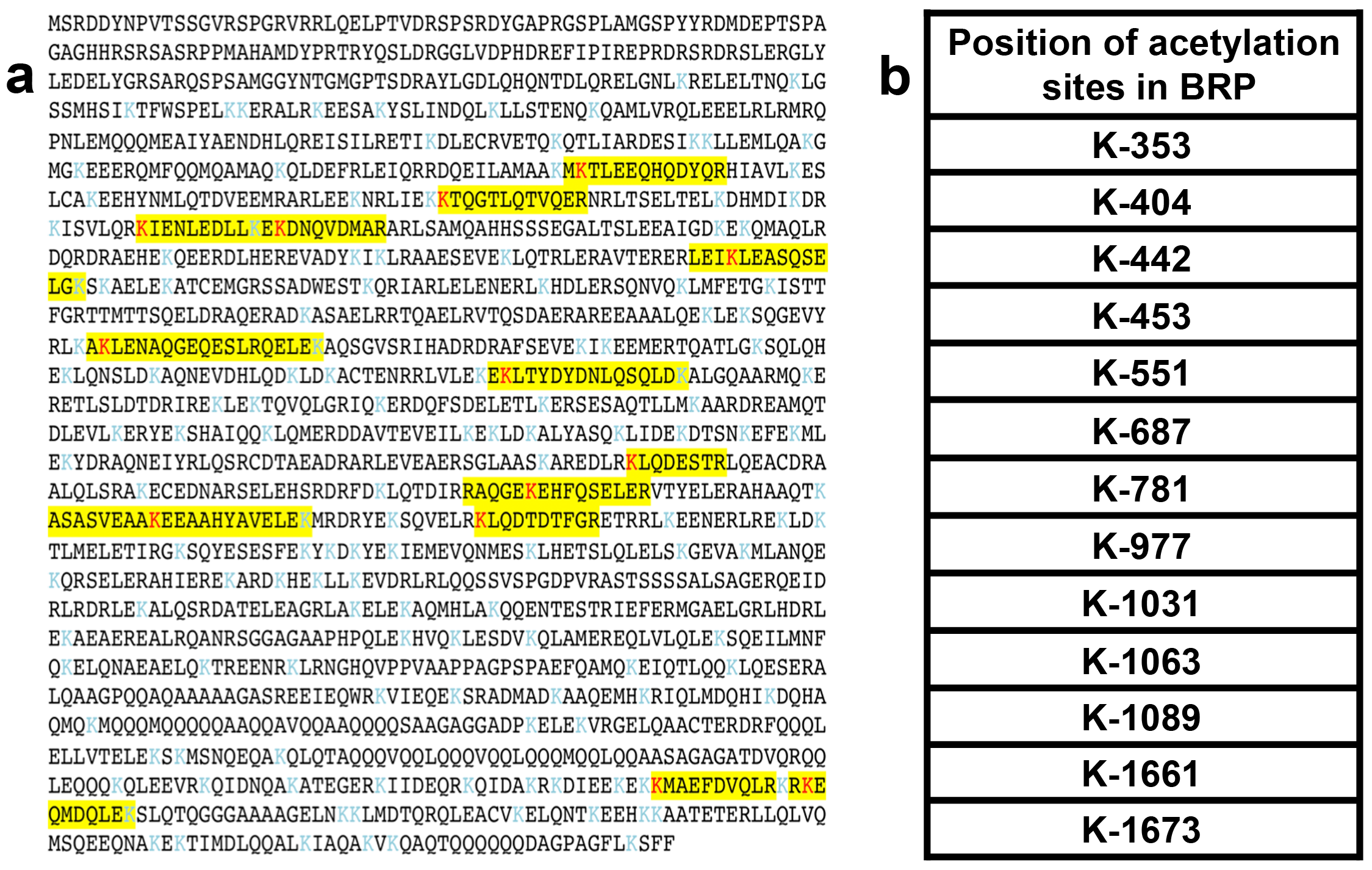

Supplement: S15 Fig — (a) BRP sequence with acetylated peptide fragments (yellow) and lysine sites positive for acetylation (red) identified through mass spectroscopy. (b) Position of possible lysine residues that undergo (de)acetylation within BRP. (TIF) [file pbio.1002563.s016.tif]

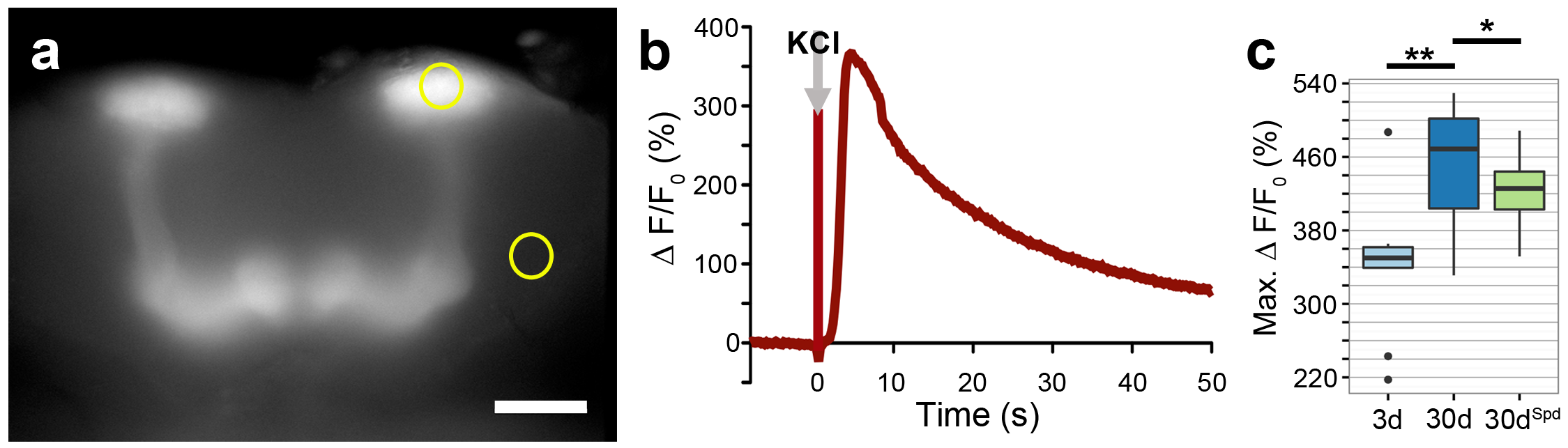

Supplement: S16 Fig — (a) Homer GCamp3.0 expressed in the dendritic claws of KCs and imaged within the calyx region. The two rings indicate the region of interest (calyx neuropil) and background region used for analysis. Scale bar: 50 μm. (b) KCl-induced influx of postsynaptic Ca2+ ion, measured by changes in fluorescence (ΔF/F in %) of Homer GCamp3.0 of a single fly over time. (c) Maximum change in fluorescence (ΔF/F in %) of Homer GCamp3.0 response to KCl in 3d, 30d, and 30dSpd flies (n = 8–9 flies; Kruskal-Wallis test). ns = not significant, p ≥ 0.05. Underlying data is shown in S1 Data. (TIF) [file pbio.1002563.s017.tif]

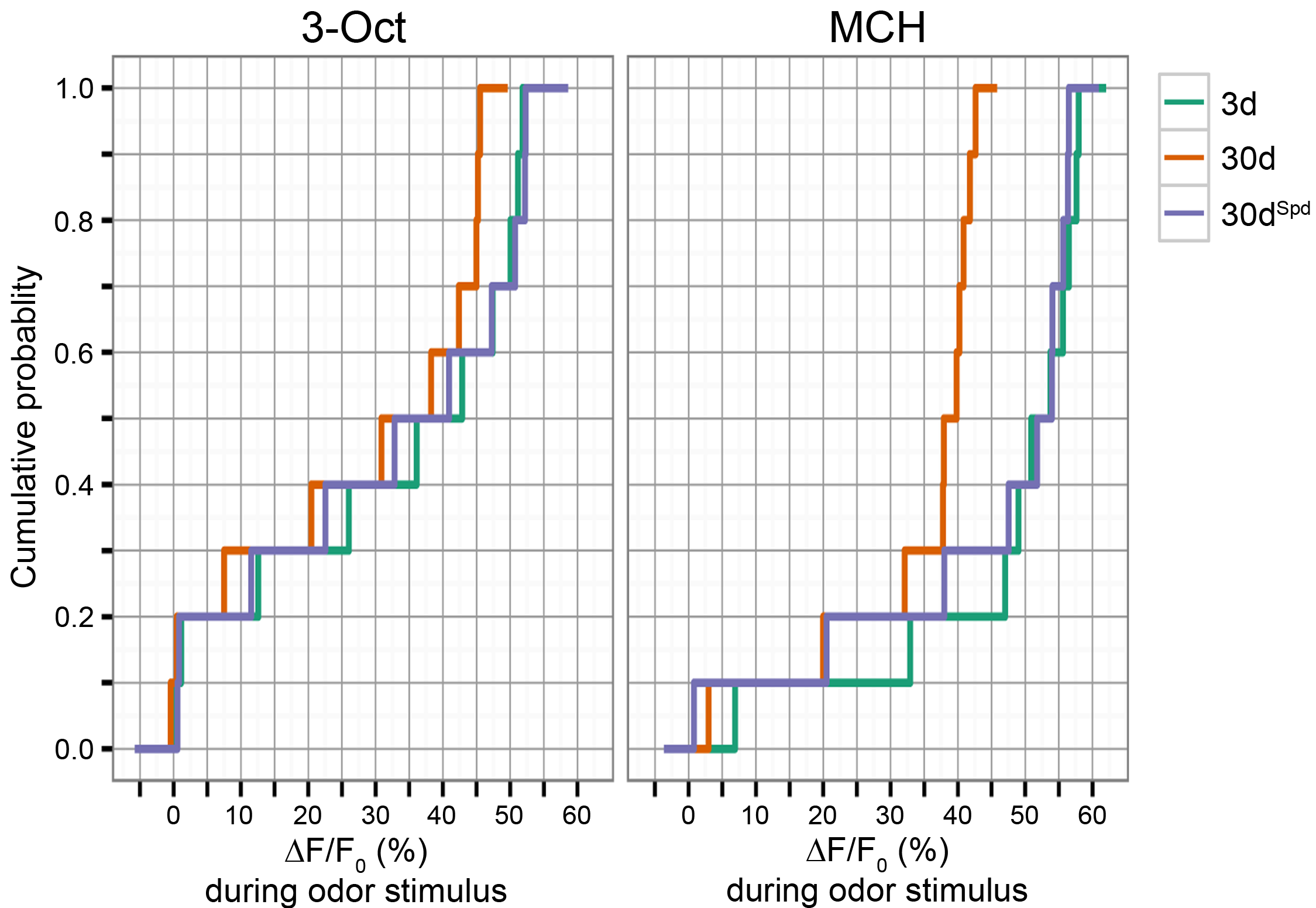

Supplement: S17 Fig — Empirical cumulative distribution functions for 3-Oct and MCH, as used in the Kolmogorov-Smirnov test. Only the GCaMP3 response during odor presentation (seconds 1–3, grey bars in Fig 3K and 3M) was used. Two-sided Kolmogorov-Smirnov tests were conducted for the analysis of difference. The differences for 3-Oct were not significant. The differences for MCH between 3d and 30d (**), as well as between 30d and 30dSpd (*) were significant after Bonferroni correction for three groups. * p < 0.05, ** p < 0.01. Underlying data is shown in S1 Data. (TIF) [file pbio.1002563.s018.tif]

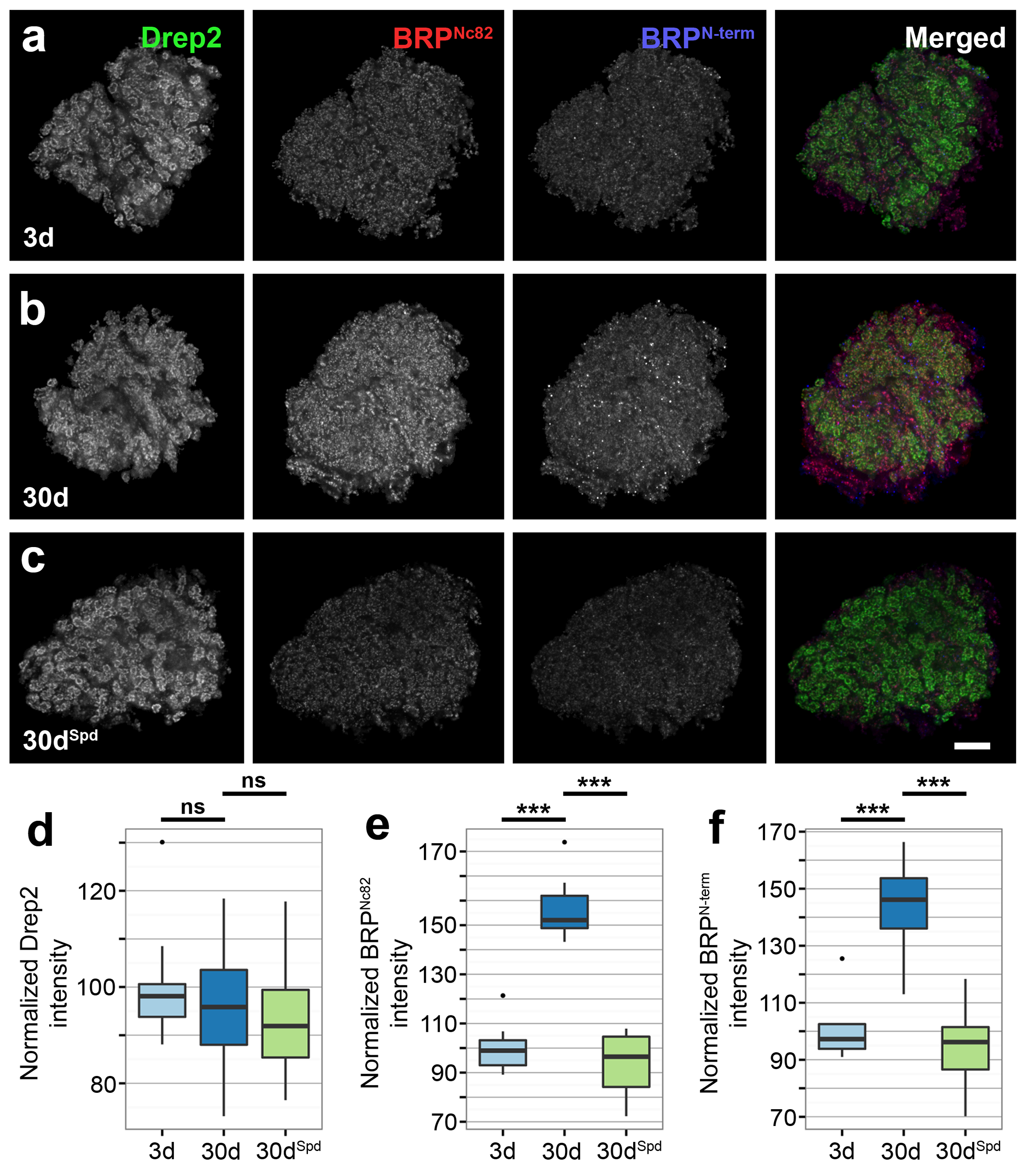

Supplement: S18 Fig — (a–c) Mushroom body calyx from adult brains of 3d and 30d w1118 flies, together with 30dSpd w1118 flies immunostained for Drep2 and BRP (using Nc82 as well as N-terminal antibodies) (corresponding single z-planes are shown). Scale bar: 10 μm. (d–f) Quantification of signal intensity of these proteins in the calyx region normalized to 3d flies (n = 10 independent brains; Kruskal-Wallis test with Dunn’s multiple comparison test, p-values were subject to Bonferroni correction). ** p < 0.01, *** p < 0.001, ns = not significant, p ≥ 0.05. Underlying data is shown in S1 Data. (TIF) [file pbio.1002563.s019.tif]

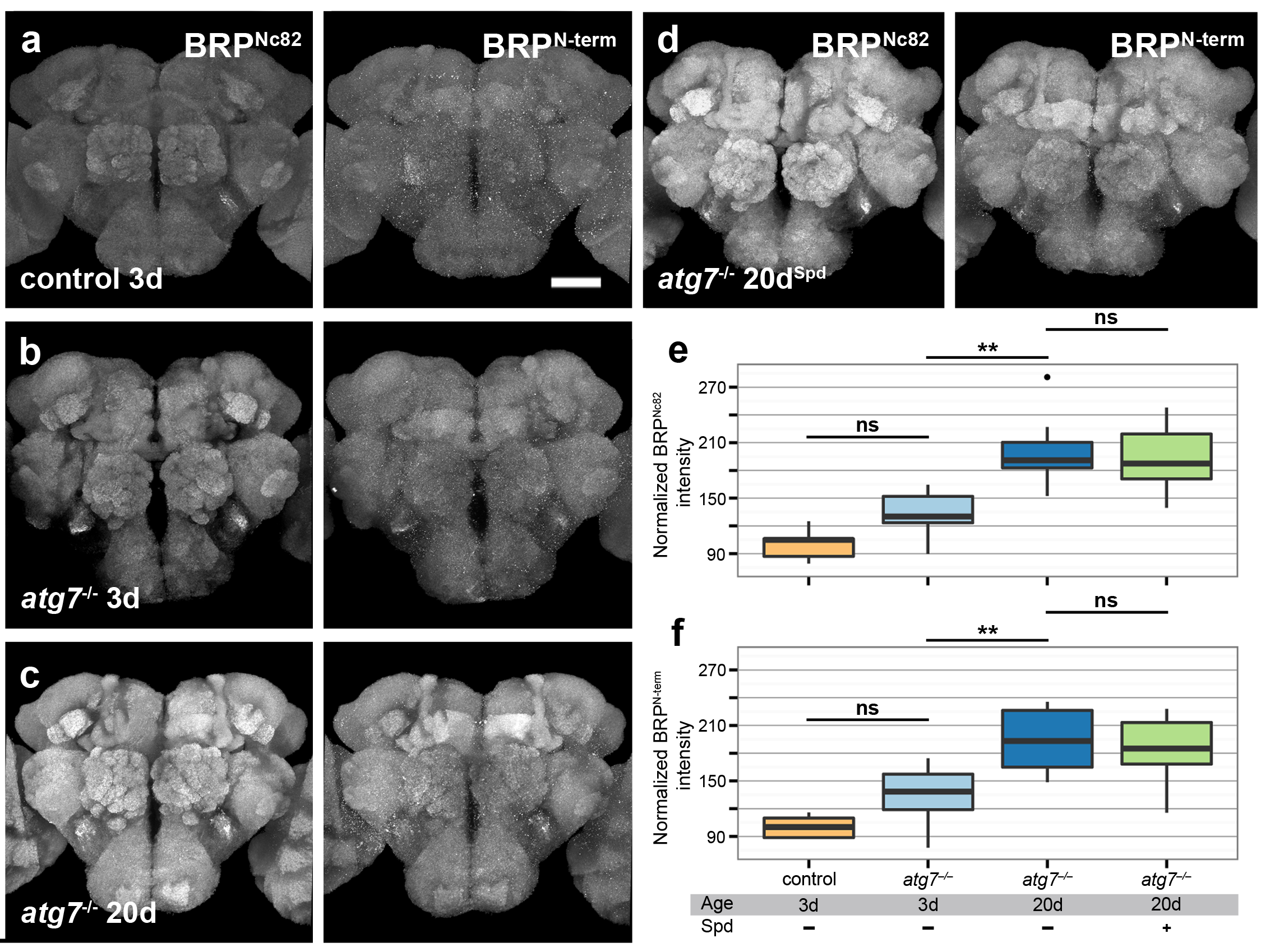

Supplement: S19 Fig — (a–d) Comparison of BRP signal intensity (using Nc82 and N-terminal antibodies) in brains of 3d w1118 control animals, 3d and 20-d old (20d) atg7-/- flies, raised either on normal or spermidine-supplemented food. Scale bar: 50 μm. (e–f) Quantification of BRP within the central brain region of young (3d) and old (20d) atg7-/- mutants normalized to 3d w1118 flies (n = 9–12 independent brains; Kruskal-Wallis test with Dunn’s multiple comparison test, p-values were subject to Bonferroni correction). ** p < 0.01, ns = not significant, p ≥ 0.05. Underlying data is shown in S1 Data. (TIF) [file pbio.1002563.s020.tif]

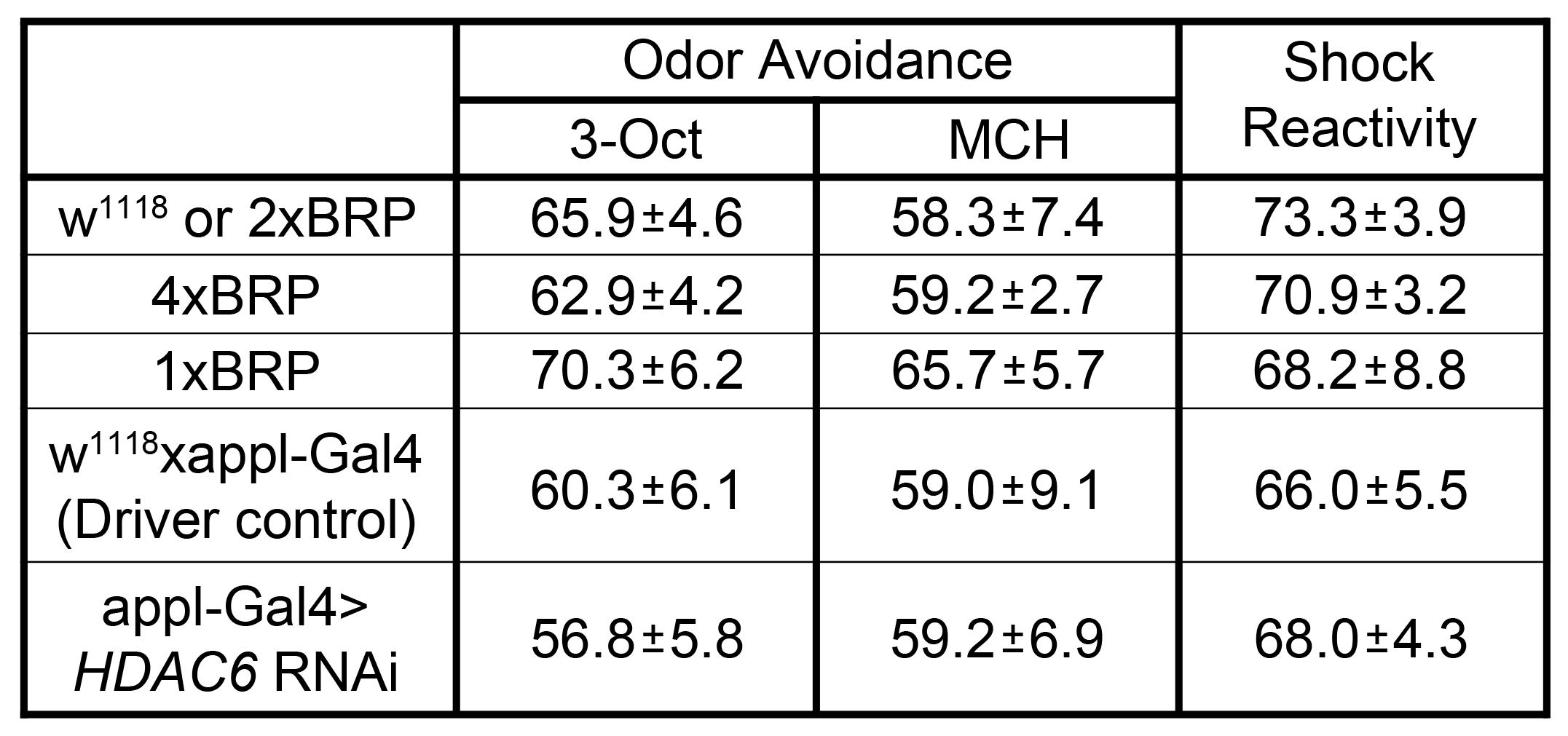

Supplement: S1 Table — Underlying data is shown in S1 Data. (TIF) [file pbio.1002563.s021.tif]
